# Comparing Transgenic Production to Supplementation of ω-3 PUFA Reveals Distinct But Overlapping Mechanisms Underlying Protection Against Metabolic and Hepatic Disorders

**DOI:** 10.1093/function/zqac069

**Published:** 2022-12-29

**Authors:** Noëmie Daniel, Mélanie Le Barz, Patricia L Mitchell, Thibault V Varin, Isabelle Bourdeau Julien, Dominique Farabos, Geneviève Pilon, Josée Gauthier, Carole Garofalo, Jing X Kang, Jocelyn Trottier, Olivier Barbier, Denis Roy, Benoit Chassaing, Emile Levy, Frédéric Raymond, Antonin Lamaziere, Nicolas Flamand, Cristoforo Silvestri, Christian Jobin, Vincenzo Di Marzo, André Marette

**Affiliations:** Faculty of Agricultural and Food Sciences, School of Nutrition, Laval University, Quebec, QC G1V 0A6, Canada; Quebec Heart and Lung Institute Research Centre, Laval University, Quebec, QC G1V 4G5, Canada; Institute of Nutrition and Functional Foods (INAF), Centre NUTRISS, Quebec, QC G1V 0A6, Canada; Quebec Heart and Lung Institute Research Centre, Laval University, Quebec, QC G1V 4G5, Canada; Institute of Nutrition and Functional Foods (INAF), Centre NUTRISS, Quebec, QC G1V 0A6, Canada; Faculty of Medicine, Department of Medicine, Laval University, QC G1V 0A6, Canada; Quebec Heart and Lung Institute Research Centre, Laval University, Quebec, QC G1V 4G5, Canada; Institute of Nutrition and Functional Foods (INAF), Centre NUTRISS, Quebec, QC G1V 0A6, Canada; Quebec Heart and Lung Institute Research Centre, Laval University, Quebec, QC G1V 4G5, Canada; Institute of Nutrition and Functional Foods (INAF), Centre NUTRISS, Quebec, QC G1V 0A6, Canada; Institute of Nutrition and Functional Foods (INAF), Centre NUTRISS, Quebec, QC G1V 0A6, Canada; Canada Excellence Research Chair on the Microbiome-Endocannabinoidome Axis in Metabolic Health (CERC-MEND), Laval University, Quebec, QC G1V 0A6, Canada; Saint Antoine Research Center, Sorbonne University INSERM UMR 938; Assistance Publique - Hôpitaux de Paris, Clinical Metabolomics department, Hôpital Saint Antoine, Paris, 75571, France; Quebec Heart and Lung Institute Research Centre, Laval University, Quebec, QC G1V 4G5, Canada; Institute of Nutrition and Functional Foods (INAF), Centre NUTRISS, Quebec, QC G1V 0A6, Canada; Department of Medicine, Department of Infectious Diseases and Immunology, and Department of Anatomy and Cell Physiology, University of Florida, Gainesville FL, 32608, USA; Department of Nutrition, University of Montreal, Montreal QC H3T 1A8, Canada and Research Centre, Sainte-Justine Hospital, Montreal, QC H3T 1C5, Canada; Laboratory for Lipid Medicine and Technology, Department of Medicine, Massachusetts General Hospital and Harvard Medical School, Charlestown MA 02129, USA; Laboratory of Molecular Pharmacology, CHU-Quebec Research Centre, and Faculty of Pharmacy, Laval University, Quebec, QC G1V 0A6, Canada; Laboratory of Molecular Pharmacology, CHU-Quebec Research Centre, and Faculty of Pharmacy, Laval University, Quebec, QC G1V 0A6, Canada; Faculty of Agricultural and Food Sciences, School of Nutrition, Laval University, Quebec, QC G1V 0A6, Canada; Institute of Nutrition and Functional Foods (INAF), Centre NUTRISS, Quebec, QC G1V 0A6, Canada; INSERM U1016, Mucosal Microbiota in Chronic Inflammatory Diseases’ Team, CNRS UMR 8104, University of Paris, Paris, 75014, France; Department of Nutrition, University of Montreal, Montreal QC H3T 1A8, Canada and Research Centre, Sainte-Justine Hospital, Montreal, QC H3T 1C5, Canada; Institute of Nutrition and Functional Foods (INAF), Centre NUTRISS, Quebec, QC G1V 0A6, Canada; Canada Excellence Research Chair on the Microbiome-Endocannabinoidome Axis in Metabolic Health (CERC-MEND), Laval University, Quebec, QC G1V 0A6, Canada; Saint Antoine Research Center, Sorbonne University INSERM UMR 938; Assistance Publique - Hôpitaux de Paris, Clinical Metabolomics department, Hôpital Saint Antoine, Paris, 75571, France; Quebec Heart and Lung Institute Research Centre, Laval University, Quebec, QC G1V 4G5, Canada; Faculty of Medicine, Department of Medicine, Laval University, QC G1V 0A6, Canada; Canada Excellence Research Chair on the Microbiome-Endocannabinoidome Axis in Metabolic Health (CERC-MEND), Laval University, Quebec, QC G1V 0A6, Canada; Quebec Heart and Lung Institute Research Centre, Laval University, Quebec, QC G1V 4G5, Canada; Institute of Nutrition and Functional Foods (INAF), Centre NUTRISS, Quebec, QC G1V 0A6, Canada; Faculty of Medicine, Department of Medicine, Laval University, QC G1V 0A6, Canada; Canada Excellence Research Chair on the Microbiome-Endocannabinoidome Axis in Metabolic Health (CERC-MEND), Laval University, Quebec, QC G1V 0A6, Canada; Department of Medicine, Department of Infectious Diseases and Immunology, and Department of Anatomy and Cell Physiology, University of Florida, Gainesville FL, 32608, USA; Quebec Heart and Lung Institute Research Centre, Laval University, Quebec, QC G1V 4G5, Canada; Institute of Nutrition and Functional Foods (INAF), Centre NUTRISS, Quebec, QC G1V 0A6, Canada; Faculty of Medicine, Department of Medicine, Laval University, QC G1V 0A6, Canada; Canada Excellence Research Chair on the Microbiome-Endocannabinoidome Axis in Metabolic Health (CERC-MEND), Laval University, Quebec, QC G1V 0A6, Canada; Joint International Research Unit on Chemical and Biomolecular Research on the Microbiome and its Impact on Metabolic Health and Nutrition between Laval University and Consiglio Nazionale delle Ricerche, Institute of Biomolecular Chemistry, Campania, 80078, Italy; Quebec Heart and Lung Institute Research Centre, Laval University, Quebec, QC G1V 4G5, Canada; Institute of Nutrition and Functional Foods (INAF), Centre NUTRISS, Quebec, QC G1V 0A6, Canada; Faculty of Medicine, Department of Medicine, Laval University, QC G1V 0A6, Canada

**Keywords:** *fat-1* gene, *Allobaculum*, nonalcoholic fatty liver disease, gut-liver axis, ω-3 PUFA, endocannabinoids

## Abstract

We compared endogenous ω-3 PUFA production to supplementation for improving obesity-related metabolic dysfunction. Fat-1 transgenic mice, who endogenously convert exogenous ω-6 to ω-3 PUFA, and wild-type littermates were fed a high-fat diet and a daily dose of either ω-3 or ω-6 PUFA-rich oil for 12 wk. The endogenous ω-3 PUFA production improved glucose intolerance and insulin resistance but not hepatic steatosis. Conversely, ω-3 PUFA supplementation fully prevented hepatic steatosis but failed to improve insulin resistance. Both models increased hepatic levels of ω-3 PUFA-containing 2-monoacylglycerol and N-acylethanolamine congeners, and reduced levels of ω-6 PUFA-derived endocannabinoids with ω-3 PUFA supplementation being more efficacious. Reduced hepatic lipid accumulation associated with the endocannabinoidome metabolites EPEA and DHEA, which was causally demonstrated by lower lipid accumulation in oleic acid-treated hepatic cells treated with these metabolites. While both models induced a significant fecal enrichment of the beneficial *Allobaculum* genus, mice supplemented with ω-3 PUFA displayed additional changes in the gut microbiota functions with a significant reduction of fecal levels of the proinflammatory molecules lipopolysaccharide and flagellin. Multiple-factor analysis identify that the metabolic improvements induced by ω-3 PUFAs were accompanied by a reduced production of the proinflammatory cytokine TNFα, and that ω-3 PUFA supplementation had a stronger effect on improving the hepatic fatty acid profile than endogenous ω-3 PUFA. While endogenous ω-3 PUFA production preferably improves glucose tolerance and insulin resistance, ω-3 PUFA intake appears to be required to elicit selective changes in hepatic endocannabinoidome signaling that are essential to alleviate high-fat diet-induced hepatic steatosis.

## Graphical Abstract

**Graphical Abstract:**
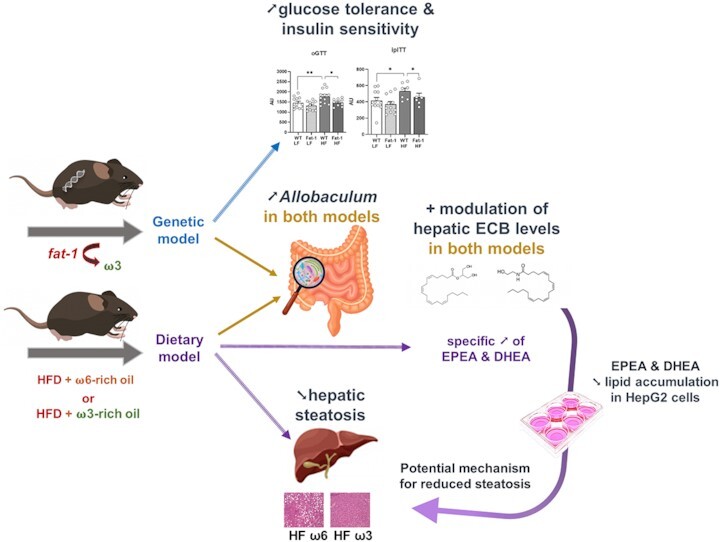

## Introduction

The gut-liver axis plays an essential role in nutrient absorption and hepatic disease pathogenesis.^1^,^2^ Nonalcoholic fatty liver disease (NAFLD) is linked with intestinal disorders including small intestine bacterial overgrowth, gut dysbiosis, and increased intestinal permeability to pathogenic bacterial factors.^3^ The gut microbiota plays a significant role in the fate of nutrients since it represents a complex interface between the luminal content and epithelial cells. Metabolites produced after digestion by enzymes from host and microbial cells can be absorbed and then reach the liver through the portal vein. Previous studies reported that ω-3 PUFA intake modulates gut microbiota populations in favor of some beneficial bacterial species.^4^,^5^ The endocannabinoidome, a complex system strongly involved in the regulation of metabolism and its pathological disturbances, is also affected by dietary ω-3 PUFA in both rodents and humans.^6–8^ Peripheral tissue or circulating levels of the endocannabinoids, anandamide (AEA), and 2-arachidonoylglycerol (2-AG)—which are derived from the ω-6 PUFA arachidonic acid (AA)—are reduced by ω-3 PUFA-enriched diets. This mechanism may underlie the protective effects of such diets on hepatic steatosis, hypertriglyceridemia, and insulin resistance in both genetic or diet-induced obesity in rodents and in obese men.^9–13^ Concomitantly, the circulating and peripheral tissue levels of ω-3 PUFA congeners of AEA and 2-AG, the ω-3 PUFA-containing *N*-acylethanolamines (NAEs) and 2-monoacylglycerols (2-MAGs), which act as anti-inflammatory mediators^14–16^ are increased following higher dietary intake of ω-3 PUFA in both rodents and humans.^8^,^17^,^18^ Interestingly, the signaling system encompassing endocannabinoids and their congeners (known as “endocannabinoidome”) is also involved in the host-gut microbiota cross-talk playing a role in obesity and NAFLD development^19^,^20^ and determines gut microbiota composition independently of body weight.^8^,^21^ The *fat-1* transgenic mouse model developed by Kang et al. is widely used to study ω-3 PUFA impact on metabolism.^22^ These animals express the *fat-1* gene from *Caenorhabditis elegans* encoding for a desaturase that converts ω-6 into ω-3 PUFA. Thus, Fat-1 mice display a reduced ω-6:ω-3 ratio, without dietary intake of ω-3 PUFA.^22^ We previously published that hemizygous *fat-1*^+/−^ mice fed a high-fat (HF) diet were protected against insulin resistance and visceral adipose tissue (VAT) inflammation without any change in food intake or body weight gain.^23^ These effects were associated with changes in VAT morphology and gene expression patterns as well as the increased biosynthesis of the ω-3 PUFA-derived lipid mediators 17-hydroxy-docosahexaenoic acid (17-HDHA) and 18-hydroxyeicosapentaenoic acid (18-HEPE), which are respectively derived from docosahexaenoic acid (DHA) and eicosapentaenoic acid (EPA).^23^,^24^ This finding supports the concept that not only ω-3 PUFAs but also their metabolites, may be metabolically beneficial, and raises the possibility that other ω-3 PUFA-derived lipid mediators, notably NAEs and MAGs are also increased and potentially participate in the above-mentioned beneficial effects. Surprisingly, the endogenous increase of ω-3 PUFA levels in the hemizygous *fat-1*^+/−^ mice did not result in prevention of HF diet-induced hepatic steatosis despite marked improvements of insulin resistance and glucose tolerance.^23^ This was unexpected given previous studies reporting that supplementation of marine ω-3 PUFA protects from hepatic fat accretion,^25^ and suggests a marked difference between the impacts of dietary vs endogenous ω-3 PUFA on hepatic steatosis.

In the present study, we wanted to further explore our previous findings, to understand this dichotomy between these 2 models by establishing how the endogenous production (genetic model) or the supplementation (dietary model) of ω-3 PUFA differentially affects HF diet-induced metabolic alterations, focusing on the gut-liver axis, and how these models modulate the gut microbiota composition. We thus performed several analyses to identify the potential mechanisms underlying the impact of ω-3 PUFA on glucose intolerance and NAFLD, with specific emphasis on potential differences resulting from exogenous vs endogenous ω-3 PUFA-tissue enrichment on hepatic lipid, inflammatory, and endocannabinoid profiles.

## Methods

### Animal Models

Hemizygous male *fat-1^+/^^−^* mice^22^ and their wild-type (WT) C57Bl/6 J littermates, were bred in-house at the Quebec Heart and Lung Institute animal facility (May 15, 2013 protocol). Seven-week-old mice were randomly assigned to five groups (*n* = 9–13/group) and housed individually in a controlled environment (12 h daylight cycles, 6 am–6 pm, 22°C), with food and water provided ad libitum. After 1 wk of acclimation on a chow diet (TD 2018), mice were fed either a low-fat [LF, TD 120651, Tecklad, 10% kcal fat (0.05% LA)–67% vegetable hydrogenated shortening, 33% corn oil, 7% kcal sucrose] or a HF diet [TD 93075, Tecklad, 55% kcal fat (3.9% LA)–95% vegetable hydrogenated shortening, 5% corn oil, 7% kcal sucrose]. Animals were gavaged daily during 12 wk with either ω-6 (safflower 0.21 mg/uL LA, President Choice) or ω-3 PUFA-rich oil (Webber Natural® capsules containing 900 mg ω-3 PUFA–600 mg EPA, 300 mg DHA) at 2.5μL/g of body weight. Doses were determined according to previous results and corresponded to ∼6% of the daily energy intake and equivalent to ∼4.6% of dietary lipids in the HF diet. Treatment groups were as follows: (1) for the Fat-1 part (genetic model): WT-LF, Fat-1-LF, WT-HF, and Fat-1-HF, all supplemented with ω-6 PUFA-rich oil; (2) for the fish oil supplementation part (dietary model): WT-LF and WT-HF supplemented with ω-6 PUFA-rich oil (LF-ω6 and HF-ω6) and WT-HF supplemented with fish oil (HF-ω3). WT-LF and WT-HF groups were used in both parts. In the dietary part, they were identified in the text as LF-ω6 and HF-ω6 and used as reference and control group, respectively, when compared with the HF-ω3 group. Energy efficiency was calculated as follows: final body weight (g)/total food intake (kcal)*100.

### Physiological Tests

At weeks 10 and 12, mice were subjected to an intraperitoneal insulin tolerance test (ipITT, 0.65 U/kg) and an oral glucose tolerance test (oGTT, 1 g/kg), respectively.^26^ After 12 wk, mice were euthanized by cardiac puncture following isoflurane anesthesia. Blood was collected in EDTA-coated tubes, centrifuged and the plasma fractions were collected and stored at −80°C. At sacrifice, tissues were collected, snap frozen in liquid nitrogen, and stored at −80°C. This study followed the Guide for the care and use of laboratory animals and all procedures had been previously approved by the Laval University Animal Ethics Committee.

### Biochemical Analyses

Insulinemia was measured using the Ultrasentitive ELISA kit (Alpco 80-INSMSU-E01, Salem NH). Hepatic triglycerides (TG) were extracted in methanol/chloroform.^26^ Plasma and hepatic TG were analyzed with Triglycerides Reagent kit (Randox Laboratories, UK). Cholesterol (Randox Laboratories, UK), nonesterified fatty acids (NEFA) (Thermo Scientific, USA) and leptin (Mouse/Rat Leptin Quantikineࣨ ELISA Kit, R&D Systems) were also measured in plasma samples. Alanine (ALT) and aspartate (AST) aminotransferases plasma levels were determined by biochemical analyses at the Quebec Heart and Lung Institute clinical biochemistry department. To determine cytokine concentrations in liver, proteins from 75 mg of tissue were extracted in PBS 1X buffer (Igepal 1% and proteinase inhibitors 1X). Proteins were measured (Pierce BCA Protein Bioassay kit) and standardized for the multiplex assay, which was performed according to the manufacturer’s instructions (Biorad). Analysis of FA profile in liver was achieved at CHU Sainte-Justine Research Center.^27^

### Endocannabinoidome Measurements

Plasma samples (40 μL) liver and muscle samples (5 to 10 mg) were extracted, and lipid mediators were measured using high-pressure liquid chromatography–tandem mass spectrometry (HPLC–MS/MS). The method can differentiate monoacylglycerol isomers at positions 1(3) and 2, but signals from both isomers of unsaturated FA were summed prior to analysis in order to account for their rapid interconversion. The following metabolites were quantitated: *N*-linoleoylethanolamine (LEA), *N*-arachidonoylethanolamine (AEA), *N*-docosahexaenoylethanolamine (DHEA), 2-linoleoyl-glycerol (2-LG), 2-arachidonoyl-glycerol (2-AG), 2-eicosapentaenoyl-glycerol (2-EPG), 2-docosapentaenoyl(n-3)-glycerol (2-DPG), and 2-docosahexaenoyl-glycerol (2-DHG).^21^

### Phospholipid Extraction and Analysis

Lipid extraction and quantification process was adapted from Lamaziere et al.^28^ and Shillito et al.^29^ Briefly, total lipids were extracted from liver tissues by the method of Folch et al.^30^ Individual phospholipid (PL) classes and their molecular species were quantified by a triple quadrupole Qtrap 6500, ABSciex (Les Ulis, France) in tandem with a Shimadzu Nexera XR liquid chromatography system (Shimadzu France, Marne la Vallee, France). A YMC-Pack PVA-Sil, bonded with a monomolecular polymer coating of vinyl alcohol (PVA) [Particle size: 5 μm, Pore size: 120 Å, Usable pH range: 2.0–9.5; (YMC, Japan)] was used to separate the different lipid classes. Concentrations of the PL compounds [phosphatidylethanolamine (PE), phosphatidylcholine (PC), and ceramide] were determined by comparing the peak area of each complex lipid with that of standards added with a known quantity.

Lipidomics: Tissues samples (25 mg) were homogenized using a bullet blender (Next Advance, NY, USA) in 300 μL of a chloroform: methanol solution (2:1) containing 0.005% of butylated hydroxytoluene (BHT), 100 mg of stainless steel 0.9–2 mm beads, and 50 μL of internal standard (Resolvin D1-d_5_, RvD1-d_5_; 1 ng/mL; Resolvin D2-d_5_, RvD2-d_5_; 5 ng/mL; leukotriene B_4_-d_4_; LTB_4_-d_4_, 5 ng/mL; DHA-d_5_, 200 ng/mL; EPA-d_5_, 200 ng/mL; 9S-hydroxyocatdecadienoic acid-d, 9S-HODE-d_4_, 1 ng/mL; Cayman chemicals, MI, USA). Homogenates were centrifuged at 4713xg rpm for 10 min; supernatants were evaporated under nitrogen to 100 μL and diluted in 3 mL of water 0.1% formic acid before performing solid phase extraction (SPE) using Strata-X 60 mg columns (Phenomenex, Torrance, CA, USA) preconditioned with methanol and water 0.1% formic acid. Solid phase extraction columns were then washed 2 times with water (2 mL) and water: methanol solution (80:20) 0.1% formic acid (2 mL). Analytes were eluted using 2 mL methyl formate. Elutes were evaporated under nitrogen, reconstituted in 100 μL of water: methanol (40:60) and filtered with microspin 0.2 μmol/L filter column (Canadian Life Science, Dorval, Canada). The same procedure was also applied to analytical standards initially diluted in PBS. Fifteen microliter was then injected into the chromatographic system consisting of an ultra-high-pressure liquid chromatography (UHPLC) instrument (Shimadzu Scientific Instruments, Columbia, MD, USA). The chromatographic separation was achieved with a C_18_ column from Agilent (150 × 2.1 mm Poroshell 120 EC; 2.7 μm particles; Santa Clara, CA, USA) at 40°C, and the following conditions: solvent A = ammonium formate in water (5 mmol/L) 0.01% acetic acid and solvent B = methanol 0.01% acetic acid. Separation was performed at a flow rate of 0.3 mL/min as follows: 60% B and linear gradient to 70.8% B over the next 12 min, B was increase to 80% over the next 7 min and increase to 85% over 5 min. Column was then flushed at 100% B during 4 min, and return to initial conditions over the next 7 min. All analytes were quantified by tandem mass spectrometry (MS/MS) using an API6500 instrument (Applied Biosystems, Concord, ON, Canada). The temperature was set at 550°C and entrance potential (EP) at −8 V. MS/MS parameters [ion transition (m/z); declustering potential (volt); and collision energy (volt)] were as follows: DHA (327.2→229.2; −80; −20); EPA (301.1→203.0; −80; −20); 17hydroxy-DHA (17S-HDHA: 343.1→201.1; −65; −20); 18R/S-HEPE (317.1→215.0; −80; −21) PDX (359.1→206.0; −60; −21); PD1 (359.1→206.0; −60; −21); RvD1 (375.1→140.9; −50; −20); 17R-RvD1 (375.1→140.9; −50; −20); RvD2 (375.1→175.0; −70; −31); DHA-d_5_ (332.2→230.2; −80; −20); EPA-d_5_ (306.1→208.0; −80; −20); 9S-HODE-d_4_ (299.3→171.9; −80; −26); LTB4-d_5_ (335.1→195.0; −75; −22); RvD1-d_5_ (380.1→140.9; −50; −20); and RvD2-d_4_ (380.1→175.0; −70; −31). Under these conditions, the limits of quantification were 1 ng for DHA and EPA; 10 pg for RvD2; 5 pg for 17S-HDHA, 18R/S-HEPE, RvD1, and 17R-RvD1; 1 pg for PDX and PD1.^24^,^31^,^32^ Where a valid peak was detected but below the level of quantification (BLOQ) the method of utilizing level of quantification/2 (LOQ/2)^33^ was employed.

### Bile Acid Measurement

Samples (25 mg) were homogenized using a bullet blender (Next Advance, NY, USA) and 100 mg of stainless-steel beads (0.9–2 mm) in 500 μL of a methanol solution (0.1% formic acid). Fifty microliters of internal standards (mix of CDCA-d4, DCA-d4, CA-d4, LCA-d4, and GCA-d4; C/D/N Isotopes Montréal, Canada) were added. Homogenates were centrifuged at 5000 *g* for 5 min. Supernatants were then evaporated under nitrogen and suspended in 1 mL of a water-0.1% formic acid solution, before performing SPE using a preconditioned (methanol and water-0.1% formic acid) Strata-X 60 mg 96-wells plate (Phenomenex, Torrance, CA, USA). Solid phase extraction columns were washed with water (2 mL) and a water: methanol solution 80:20 (v/v) containing 0.1% formic acid (2 mL). Analytes were then eluted using 2 mL methanol. Eluates were evaporated under nitrogen and reconstituted in 100 μL water: methanol 50:50 (v/v) prior injection to the LC–MS system. One microliter of each sample or analytical standard was then injected into the chromatographic system consisting of a Nexera UHPLC instrument (Shimadzu Scientific Instruments, Columbia, MD, USA). The chromatographic separation was achieved with a C18 column from Agilent (150 × 2.1 mm Poroshell 120 EC-C18; 2.7 μm particles; Santa Clara, CA, USA) at 37°C, and the following mobile phases: solvent A = ammonium acetate in water (5 mm) and solvent B = acetonitrile. Separation was performed at a flow rate of 0.3 mL/min using the following sequence: 67% A:23% B as initial conditions, then a linear gradient to 25% B over the next 10 min, followed by an increase of B to 35% in 20 min and to 60% in 10 min. Column was then flush at 95% B over the next 12 min and back to initial conditions for 8 min. All analytes were quantified by tandem mass spectrometry (MS/MS) using an API6500 instrument (Applied Biosystems, Concord, ON, Canada). The temperature was set at 500°C.

### Short-Chain Fatty Acids from Caecum

Protocol for short-chain fatty acid (SCFA) extraction in the caecum was adapted from Garcia-Villalba et al.^34^ Briefly, 100 mg of caecal content were accurately weighted and homogenized in 1 mL of phosphoric acid 0.5% containing 4-Methylvaleric acid (diluted in ethyl acetate and used as intern standard). After 10 min of centrifugation, 17 949 × g at 4°C, supernatants were transferred in a new 1.5 mL conic tube with 1:1 (v/v) of ethyl acetate and vortexed during 2 min. Two aliquots of volume 200 µL each were stored at –20°C until analysis by gas chromatography-flame ionization detector (GC-FID).

### Liver Histology

For structural analysis of liver, paraffin-embedded tissues were sectioned to a thickness of 4–5 mm and fixed to glass slides. Once deparaffinized, slides were stained with H&E.

### RNA Extraction from Liver and Real-Time Qpcr

Total RNA, from 25 mg liver tissue, was homogenized in TRIzol reagent (Thermo Fisher Scientific) using a VWR beadmill and purified using the Direct-zol RNA MiniPrep kit (Zymo Research). The concentration and purity (A260/280) of the RNA samples were determined on a BioTech BioDrop. One microgram total RNA was reversed transcribed using the high-capacity cDNA Reverse Transcription Kit (Thermo Fisher Scientific). Primers (IDT) were optimized for efficiency and suitability as reference genes (*Actb* and *Ppia*) and are listed in Table S5. Real-time qPCR was performed using a CFX 364 (BioRad) and results were analyzed using CFX Maestro software (BioRad).

### DNA Extraction from Feces

Bacterial DNA was extracted from fresh feces. Briefly, feces (30–50 mg) were homogenized in a lysis buffer containing lysozyme and incubated at 37°C, 30 min. Then, proteinase K and SDS 10% were added to the mix, and incubated at 60°C, 30 min. After 2 extractions with phenol:chloroform:isoamylalcohol [25:24:1 (v/v/v)] and phenol:chloroform [25:24 (v/v)], DNA was precipitated by addition of 100% EtOH in combination with 3 M sodium acetate at −20°C for 1 h. Finally, after centrifugation, precipitated DNA was re-suspended in Tris buffer (10 mm, pH8), and purified with Blood and Tissue Kit (Qiagen), according to the manufacturer’s instructions. DNA yield was assessed using a NanoDrop ND-1000 spectrophotometer (Thermo Scientific). Extracted DNA was stored at −80ºC until further use.

### 16S rRNA Gene-based Analysis

16S rRNA amplification of the V3–V4 region followed by sequencing were performed at the IBIS (Institut de Biologie Intégrative et des Systèmes—Laval University) platform.^35^ Already-demultiplexed reads were then analyzed using the QIIME software package (version 1.9.1). Paired-end sequences were merged with at least a 50-bp overlap. Downstream sequence analysis involves removing low-quality reads (Phred score ≤ 25; presence of ambiguous bases «N») and sequences with minimum and maximum lengths ≤ 400 pb and ≥ 460 pb, respectively. Forward and reverse primers were trimmed from the filtered reads, followed by detection and removal of chimeric sequence artefacts, which was performed using UCHIME.^36^ The resulting sequences were clustered into OTUs (Operational taxonomic units) using an open-reference methodology performed with USEARCH 61 version 6.1.544.^37^ Representative OTU sequences were assigned taxonomy against the Greengenes reference database (August 2013 release)^38^ using the naive Bayesian RDP-classifier.^39^ Low confidence OTUs (singletons and OTUs with a number of sequences < 0.005% of total number of sequences) were discarded.^40^ Rarefaction, to a subsampling depth of 5887 reads per sample (determined by the minimum number of sequences in a sample from a single time point) was performed on all samples of the dataset to normalize sampling effort. The RDP classifier against the RDP database (version September 30, 2016)^41^ was used to further classified OTUs that were unassigned against Greengenes at the genus level.

### Fecal Flagellin and Lipopolysaccharide Load Quantification

Levels of fecal bioactive flagellin and lipopolysaccharide (LPS) were quantified^42^ using human embryonic kidney (HEK)-Blue-mTLR5 and HEK-Blue-mTLR4 cells, respectively (Invivogen, San Diego, CA, USA). Fecal material was resuspended in PBS to a final concentration of 100 mg/mL and homogenized for 10 s using a Mini-Beadbeater-24 without the addition of beads to avoid bacteria disruption. Samples were then centrifuged at 8000 *g* for 2 min, and the resulting supernatant was serially diluted and applied on mammalian cells. Purified *Escherichia coli* flagellin and LPS (Sigma-Aldrich) were used for standard curve determination using HEK-Blue-mTLR5 and HEK-Blue-mTLR4 cells, respectively. After 24 h of stimulation, the cell culture supernatant was applied to QUANTI-Blue medium (Invivogen) and the alkaline phosphatase activity was measured at 620 nm after 30 min.

### Analysis of Lipids and Endocannabinoids Following Ω-3 PUFA Treatment in HepG2 Cells

HepG2 cells were cultured in DMEM (Multicell) supplemented with 10% fetal bovine serum (FBS) at 37°C in 5% CO. Cultures were passaged with 0.05% Trypsin-EDTA at 80% confluence.

For determination of ω3-PUFA and derived endocannabinoid concentrations cells were plated into 6-well plates at 800 000 cells per well and left to adhere for 24 h. Cells were rinsed with HBSS, and treatment conditions applied: DMEM + 10% FBS (Control), DMEM + 10% FBS + 25 µm ω-3 PUFA mix (2:1 EPA:DHA) or 100 µm ω-3 PUFA mix (2:1 EPA:DHA). After 24 h, media and cells were collected and mixed with an equal volume of methanol with 0.01% acetic acid. A Bligh and Dyer for LC–MS method was used to extract lipids.

For lipid accumulation, experiments cells were plated into 48-well plates at 100 000 cells per well and left to adhere for 24 h. Cells were rinsed with HBSS and treatment conditions applied: DMEM + 10% FBS (untreated reference), DMEM + 10% FBS + 200 µm OA in DMSO (Control); the OA media was then supplemented with one: 100 µm ω3-PUFA mix (2:1 EPA:DHA), 50 nm DHEA or 50 nm EPEA and left for 24 h. Lipid accumulation was determined using AdipoRed following the manufacturer’s instructions. Briefly, cells were rinsed with PBS, then 0.2 mL PBS + 6 µL AdipoRed was added to each well. The plate was mixed and incubated for 10 min. Fluorescence was determined using a BioTek Cyt5 plate reader with excitation of 485 nm and emission at 572 nm. Lipid accumulation experiments were repeated four times in triplicate.

### Statistical Analysis

Data are expressed as mean ± SEM. For the Genetic model, statistical analysis was performed using a two-way ANOVA or a mixed model for repeated measurement analyses followed by Tukey’s post-hoc test. Data were log-transformed when violating the ANOVA postulates. Main effects (cut-off value at *P* = .10) for diet (D), gene (G), or time (T) factors and corresponding interaction effects were indicated under the title of each graph. For the Dietary model, a one-way ANOVA was performed followed by Dunnett’s post-hoc test, considering the HF-ω6 group as control. The corresponding nonparametric Kruskal–Wallis test followed by Dunn’s post-hoc test was used when data violated the ANOVA postulates. LF-ω6 group was considered as a reference for the HF-ω6 group and represented by a dotted line in bar graphs. For cell AdipoRed assay, oleic acid (OA) group was compared to Control through a two-tailed *t*-test, while a one-way ANOVA followed by Dunnett’s post-hoc test was performed to compare ω-3 PUFA and derivative treatments to OA. For endocannabinoid dosage in cells, a one-way ANOVA was performed. Significant differences (detected by post-hoc tests when *P* < .05) were recorded as follows: *P < .05, **P < .01, ***P < .001; with an exception for cell results where Control vs OA: ^###^*P* < .001. Multiple factor analysis (MFA) was made using MFA function of the “FactoMineR” package and performed using R studio software. Permutational multivariate analysis of variance (PERMANOVA) have been made using Adonis function of the package “vegan” with 100 000 permutations. Multiple factor analysis plots were made with fviz_mfa() of the factoextra package and “ggplot2” package.

## Results

### Body Weight Gain and Energy Efficiency are Improved in Both Models of Ω-3 PUFA Enrichment

**Figure 1.**
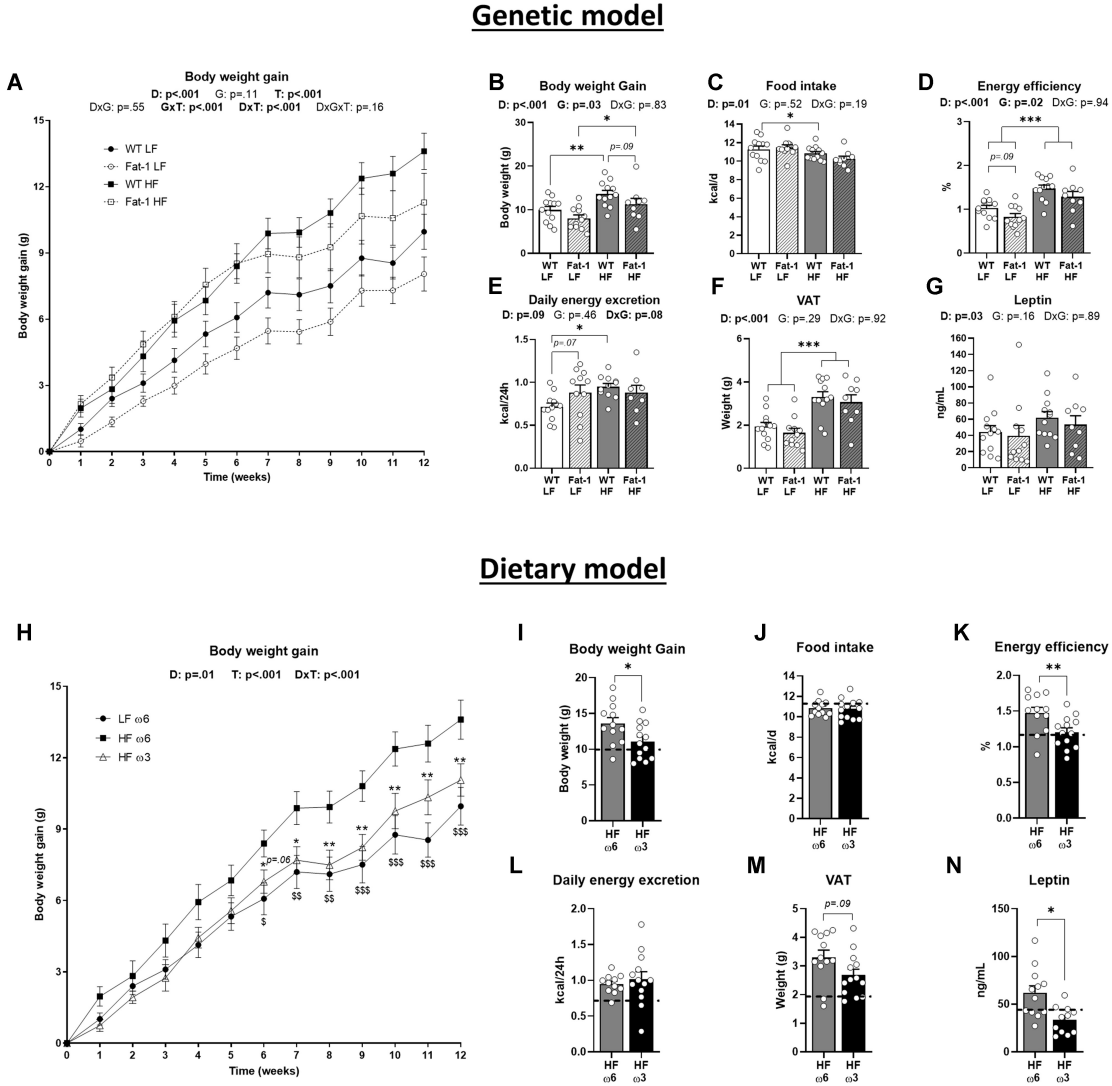
Both ω-3 PUFA supplementation and endogenous ω-3 PUFA production improved body weight gain and energy efficiency. Panels (**A–G**) refer to the genetic model and (**H**–**N**) to the dietary model. (**A, H**) Body weight gain excursion curve and (**B, I**) total body weight gain. (**C, J**) Daily food intake, (**D, K**) energy efficiency, and (**E, L**) daily energy excretion. (**F, M**) Visceral white adipose tissue weight. (**G, N**) plasma leptin. Values are means ± SEM of *n* = 9–13 mice per group. For the genetic model: two-way ANOVA followed by Tukey’s post-hoc test. *P*-values of main effects for diet (D) and gene (G) or diet × gene (D × G) interaction are recorded under the title of each graph. For the dietary model: one-way ANOVA followed by Dunnett’s post-hoc test compared to HF-ω6 group. Dotted line represents LF-ω6 group as a reference. *P < .05, **P < .01, ***P < .001.

We first analyzed obesity-related phenotypic parameters and found a global genotype effect on body weight between Fat-1 mice and their WT littermates, leading to a lower final body weight with no effect on food intake. However, it did not reach significance in the Fat-1-HF mice compared with WT mice (*P* = .09) (Figure 1A–C). We also observed a genotype effect on energy efficiency that was decreased in Fat-1 mice (Figure 1D). The daily energy excretion, the VAT mass, and the plasma leptin levels were only affected by diet in the genetic model (Figure 1E–G).

Conversely, ω-3 PUFA supplementation prevented body weight gain induced by the HF diet, and it was statistically different from week 6 (Figure 1H). At week 12, HF-ω3 mice displayed a lower body weight gain (*P* < .05), a similar food intake, and a decreased energy efficiency (*P* < .01) compared with HF-ω6 (Figure 1I–K). Daily energy excretion was not affected by ω3-rich oil gavage; the decrease of VAT accumulation did not reach significance (*P* = .09) but circulating leptin levels were reduced (*P* < .05) in HF-ω3 compared with HF-ω6 mice (Figure 1L–N).

### The Endogenous Production of Ω-3 PUFA in Fat-1 Mice Improves Insulin Sensitivity and Glucose Tolerance

**Figure 2.**
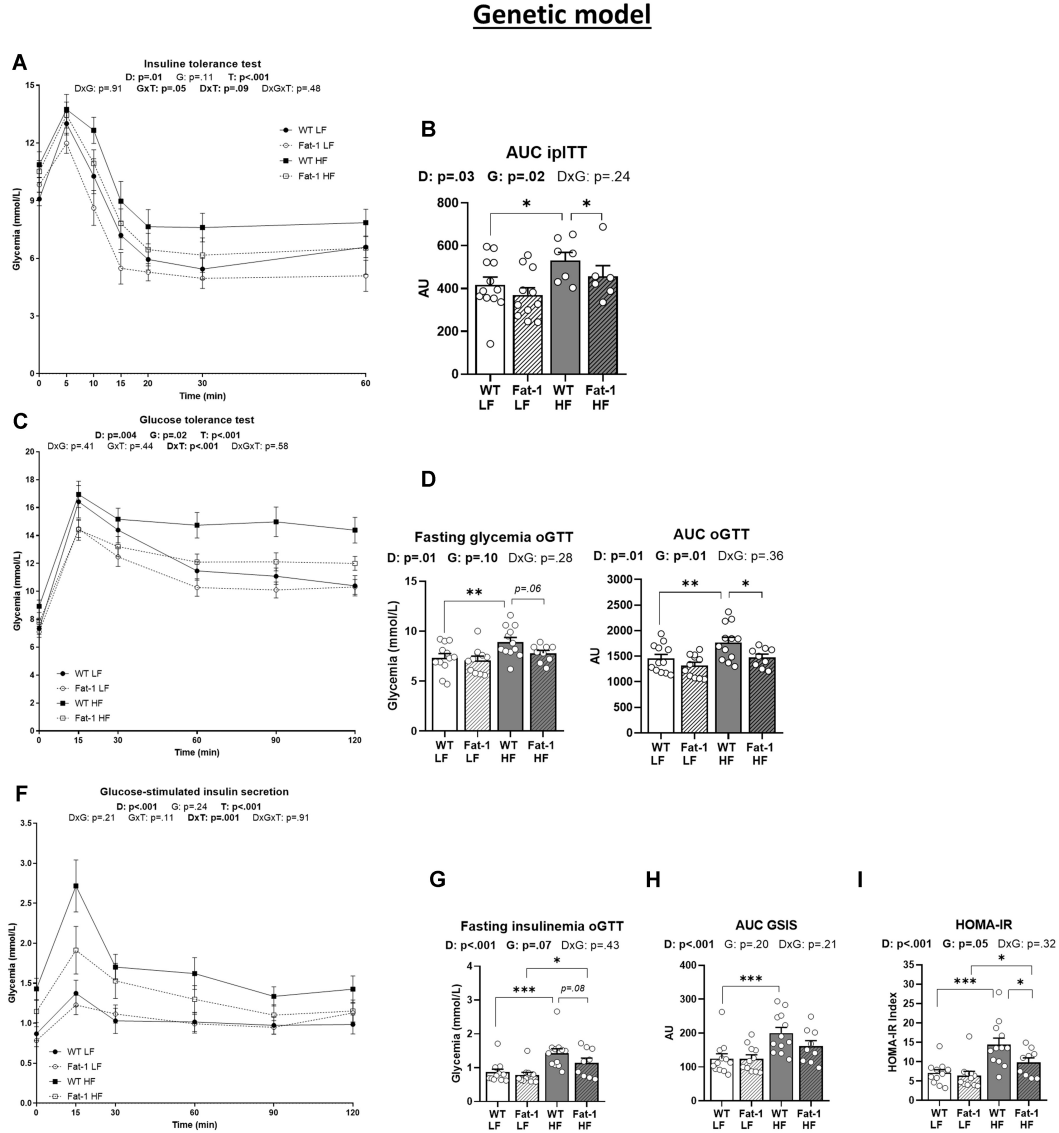
The endogenous production of ω-3 PUFA significantly improves glucose homeostasis. Panels (**A**) Glycemic response and (**B**) corresponding area under the curve (AUC) for the insulin tolerance test (ipITT) performed at week 10. (**C**) Glycemic response, (**D**) fasting glycaemia, and (**E**) AUC before and during the glucose tolerance test (oGTT) performed at week 12. (**F**) Glucose-stimulated insulin secretion, (**G**) fasting insulin, and (**H**) AUC before and during the oGTT. (**I**) Homeostasis model assessment of insulin resistance (HOMA-IR). Values are means ± SEM of *n* = 9–13 mice per group. For the genetic model: two-way ANOVA followed by Tukey’s post-hoc test. *P*-values of main effects for diet (D) and gene (G) or diet × gene (D × G) interaction are recorded under the title of each graph. *P < .05, **P < .01, ***P < .001.

We next determined the impact of endogenous production vs ω-3 PUFA supplementation on glucose homeostasis. During the ipITT, the improvement of glucose response failed to reach significance in both Fat-1 groups compared with their WT littermates (genetic effect, *P* = .11) (Figure 2A). However, the corresponding AUC decreased in Fat-1-HF mice compared with their WT controls (*P* < .05) (Figure 2B). At 12 wk, the oGTT demonstrated that glucose tolerance was improved in Fat-1 mice (genetic effect, *P* = .02), regardless of diet (Figure 2C). The corresponding AUC were reduced in Fat-1-HF mice compared with WT-HF mice (*P* < .05) and associated with a lower fasting glycaemia (*P* = .06) (Figure 2D and E). Despite improved glucose response, the reduction of insulin secretion following the glucose bolus (GSIS) failed to reach significance (*P* = .07), the HOMA-IR index decreased significantly in Fat-1-HF compared with WT-HF mice (*P* < .05) (Figure 2F–I). On the contrary, ω-3 PUFA supplementation did not improve glucose homeostasis as shown by lack of change in fasting glycaemia, insulin sensitivity, glucose tolerance, GSIS, and HOMA-IR (Figure S1A–I).

### Both Models of Ω-3 PUFA-enrichment Modify the Gut Microbiota Composition Increasing *Allobaculum* Genus Relative Abundance

**Figure 3.**
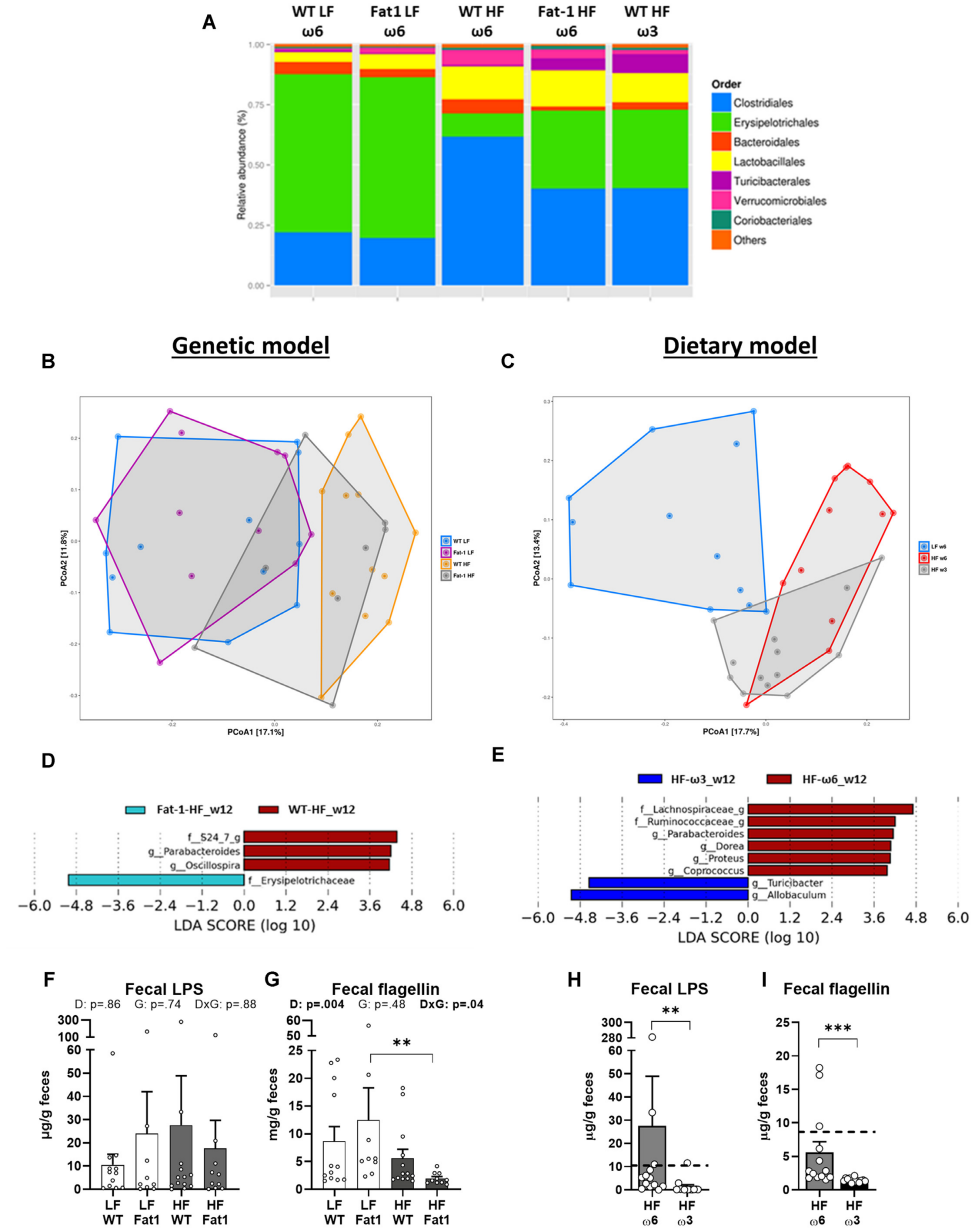
The endogenous production and supplementation of ω3-PUFA similarly modifies the gut microbiota profile in feces. (**A**) Bacterial relative abundance at the order level of the five treated groups after 12 wk of treatment. (**B−C**) Principal Coordinate Analysis (PCoA) based on unweighted Unifrac distance matrix for (**B**) genetic model (coordinate 1 and 2 accounting for 17.1% and 11.8% of the variance, respectively) and (**C**) dietary model (coordinate 1 and 2 accounting for 17.7% and 13.4% of the variance, respectively). LEfSe analysis identifying significant differentially abundant genera in (**D**) genetic model between Fat-1-HF and WT-HF mice and (**E**) dietary model between HF-ω3 and HF-ω6 mice. “g” at the end of taxon denotes unclassified genus. Fecal levels of lipopolysaccharide (LPS) and flagellin (FliC) in the genetic model (**F, G**, respectively) and the dietary model (**H, I**, respectively). Values are means ± SEM of *n* = 9–13 mice per group. For the genetic model: two-way ANOVA followed by Tukey’s post-hoc test. *P*-values of main effects for diet (D) and gene (G) or diet × gene (D × G) interaction are recorded under the title of each graph. For the dietary model: one-way ANOVA followed by Dunnett’s post-hoc test compared to HF-ω6 group. Dotted line represents LF-ω6 group as a reference. **P < .01, ***P < .001.

Once ingested, food compounds directly interact with the gut microbiota. We thus tested whether increasing ω-3 PUFA bioavailability through digestive or genetic pathways could prevent gut microbiota alterations induced by HF diet consumption. Here, HF diet induced an expected shift in the gut microbiota populations compared with LF diet-fed animals (Figure 3A; at time 0, all mice were under regular chow diet, Figure S2A), which was characterized by a significant increase in relative abundance in *Parabacteroides, Oscillospira,Dorea, Oscillibacter, Akkermansia, Ruminococcus, Streptococcus, Lactococcus*, and some genera from the *Clostridiales* order, accompanied by an important depletion in *Allobaculum* (belonging to the *Erysipelotrichales* order) and *Bifidobacterium* (Figure S2B). Interestingly, compared with control, both models of ω-3 PUFA enrichment improved the gut microbiota composition by increasing specific bacterial groups that belong to the *Erysipelotrichales* and *Turicibacterales* orders, and decreasing the proportion of *Clostridiales* and *Bacteroidales* (Figure 3A). Both models of HF-fed mice cluster separately from the corresponding LF diet-fed groups on a PCoA; the HF-Fat-1 and HF-ω3, are intermediary to these clusters (Figure 3B and C).

Analysis performed at family and genus levels revealed that ω-3 PUFA supplementation had a higher impact on gut microbiota composition than the genetic *fat-1* model. Focusing on similarities, results demonstrated a decreased relative abundance of *Parabacteroides* family in both models (Figure 3D and E). The relative abundance of a genus from the *S24-7* family (recently renamed as *Muribaculaceae* family)^43^ and *Oscillospira* were depleted in Fat-1-HF mice compared with WT-HF control (Figure 3D). Besides, ω-3 PUFA supplementation induced a reduction of *Lachnospiraceae_g, Ruminococcaceae_g, Dorea, Proteus*, and *Coprococcus* relative abundance and an increase in the *Turicibacter* and *Allobaculum* genera and (Figure 3E), the latter one being of great interest. Indeed, the major effect of both models was the similar increase of the *Erysipelotrichaceae* family, mainly represented by the *Allobaculum* genus (around 90% of the family), which was found to be significantly increased in the dietary ω-3 PUFA model (*P* < .05) but not in Fat-1-HF mice (*P* = .05; Figure 3D and E).

Fecal levels of bioactive LPS and flagellin were measured after 8 wk of treatment via cells engineered to express TLR4 and TLR5, respectively (Figure 3F–I). Results showed that endogenous ω-3 PUFA production had no significant impact on the concentration of these proinflammatory molecules (Figure 3F–G). In stark contrast, exogenous ω-3 PUFA administration through dietary exposure clearly impacts microbiota function, as revealed by the highly significant decreased fecal levels of LPS and flagellin (Figure 3H–I). Further analysis demonstrated that the endogenous production of ω-3 PUFA did not affect fecal lipocalin 2 (Lcn2), caecum weight, or caecal SCFA (Figure S3C–K), whereas caecum of HF-ω3 mice were heavier than HF-ω6 mice, with higher concentrations of isovalerate and isobutyrate (Figure S2L–T).

### Supplementation with Ω-3 PUFA Markedly Improves TG and PL Species Reducing Hepatic Steatosis

**Figure 4.**
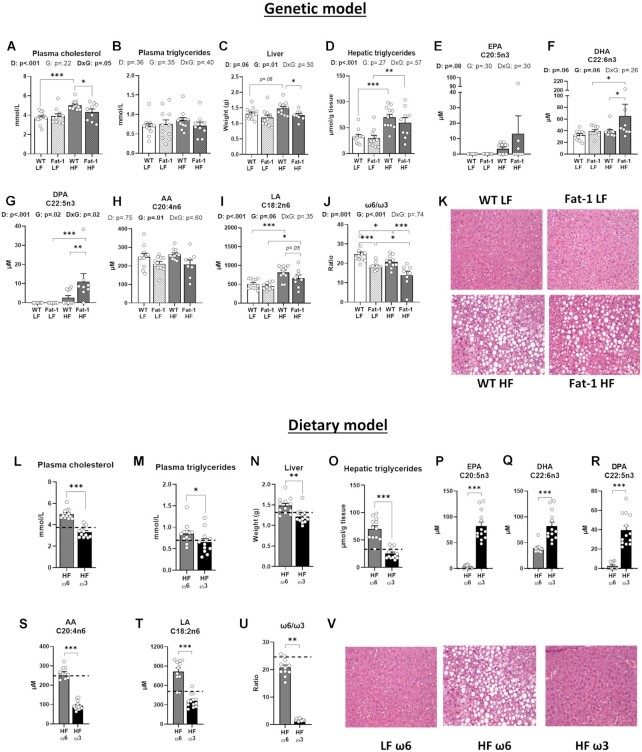
ω-3 PUFA supplementation exert a greater effect improving lipemia and reducing hepatic steatosis compared to *fat-1* endogenous production. Panels (**A–K**) refer to the genetic model and (**L**–**V**) to the dietary model. Plasma cholesterol (**A, L**) and plasma triglycerides (**B, M**). (**C, N**) Liver weight. Hepatic concentrations of (**D, O**) triglycerides, (**E, P**) EPA, (**F, Q**) DHA, (**G, R**) DPA, (**H, S**) AA, (**I, T**) LA, (**J, U**) ω6/ω3 ratio, and (**K, V**) representative hematoxylin and eosin-stained liver sections. Values are means ± SEM of *n* = 9–13 mice per group. For the genetic model: two-way ANOVA followed by Tukey’s post-hoc test. *P*-values of main effects for diet (D) and gene (G) or diet × gene (D × G) interaction are recorded under the title of each graph. For the dietary model: one-way ANOVA followed by Dunnett’s post-hoc test compared to HF-ω6 group. Dotted line represents LF-ω6 group as a reference. *P < .05, **P < .01, ***P < .001.

Knowing that the gut microbiota is closely linked to the liver through the portal vein and the importance of the gut-liver axis in metabolic health, we next investigated the impact of the 2 models on both circulating and hepatic lipid profiles. First, the determination of plasma lipid profiles revealed that Fat-1-HF mice displayed a lower cholesterol concentration compared with WT-HF mice (*P* < .05) (Figure 4A) but not circulating TG (Figure 4B), or NEFA (Figure S3A). Hepatic transaminases, nonexclusive indicators of liver injury, revealed a diet effect with ALT, but not AST, levels being significantly increased in Fat-1-HF compared with their WT littermates (Figure S3B–C). In the Fat-1 model, we found no genetic or HF diet-induced difference in EPA levels (Figure 4E). However, there was both a HF diet and Fat-1 genetic effect, which increased the DHA and docosapentaenoic acid n-3 (DPA) levels (Figure 4F–G). The HF diet effect increased the ω-6 PUFA linoleic acid (LA) (*P* = .05), while Fat-1 genetic effect decreased its concentrations. The genetic effect decreased AA (genetic effect, *P* = .01) compared with WT controls (Figure 4H–I). The detailed FA profile (Tables S1) exhibited a significant reduction of total ω-6 PUFA (*P* < .05) associated with the increase of total ω-3 PUFA (*P* < .05) in Fat-1-HF mice compared with WT-HF mice resulting in a reduced ω-6/ω-3 ratio (*P* < .001), which was also observed in the Fat-1-LF group (*P* < .001) (Figure 4J). However, at the tissue level, increased hepatic production of ω-3 PUFA in *fat-1^+/^^−^* mice did not prevent hepatic lipid accumulation (Figure 4K), as reported previously.^23^

In contrast, the supplementation of ω-3 PUFA significantly decreased plasma TG (*P* < .05) and cholesterol (*P* < .001) (Figure 4L, M), while it tended to decrease plasma NEFA (*P* = .08), ALT (*P* = .06), and AST (*P* = .07) (Figure S3D–F). In the liver, ω-3 PUFA supplementation completely inhibited ectopic fat accumulation compared with HF-ω6 control, decreasing liver weight (*P* < .01) and drastically reducing (−62%) hepatic TG (*P* < .001) (Figure 4N–O). In the dietary model, we found a higher concentration of hepatic ω-3 EPA, DHA, and DPA and a decrease in ω6 AA and LA in HF-ω3 compared with HF-ω6-treated mice (*P* < .001 for all these FA) (Figure 4P–T). Detailed lipid profile analysis (Table S1) revealed a decreased total ω-6 PUFA level (*P* < .001) and an increased total ω-3 PUFA level (*P* < .01), resulting in an ω-6/ω-3 ratio 12 times lower in HF-ω3 mice than in HF-ω6 mice (*P* < .01) (Figure 4U).

Due to their high PUFA content (at the *sn*-2 position), plasmalogens are considered key PUFA storage sites. Ether lipids are highly abundant molecules that account for approximately 20% of the total phospholipid content in mammalian cells.^44^ We thus determined the hepatic levels of several markers of interest: phosphatidylcholine (PC) species, phosphatidylethanolamine (PE) species, total PC/total PE ratio, (Tables S2 and S3, Figure S4), and total ceramides (Figures S5 and S6). The hepatic levels of ether-linked PC and PE were higher after ω-3 PUFA tissue enrichment in both models (Figure S4), with a specific ω-3 PUFA increase in long-chain PE and PC 40:6 and 40:7 species (Tables S2 and S3). The ratio of total PC/PE (Figure S4C and F) a specific marker reported as inversely correlated with hepatic steatosis development^45^ was also increased after both endogenous production and exogenous supplementation of ω-3 PUFA. These PL PUFAs can be further metabolized into potent second messenger molecules, such as protectins and resolvins, to induce anti-inflammatory or proresolving effects. We restricted our measurements to those from our previous *fat-1* animal study^23^ and for which we have developed protocols.^24^,^31^ Liver tissue was analyzed for DHA-derived 17-HDHA, Resolvin D1, Resolvin D2, protectin D1, and DX along with the EPA-derived 18-HEPE. In the genetic model, both 17-HDHA and 18-HEPE were readily detected and quantified. Endogenous production of ω-3 PUFA tended to enhance the levels of these mediators in the liver of Fat-1-LF-fed mice but not in Fat-1-HF-fed animals (Figure S4D and E), while the downstream resolvins and protectin D1 were not detectable in WT animals. Protectin DX was detectable in Fat-1-LF-fed but not Fat-1-HF-fed mice but at a concentration BLOQ. Exogenous supplementation was more effective at augmenting the concentration of 17-HDHA and 18-HEPE mediators (Figure S4I and J). Similar to the genetic model, dietary supplementation did not lead to detectable levels of resolvins or protectin-D1 but did result in detectable levels of protectin, DX which were significantly increased compared to WT-HF (Figure S4K).

Ceramides, which are biologically active lipids, link nutrient excess to mitochondrial dysfunction and oxidative stress contributing to a proinflammatory insulin resistant environment and the development of steatosis.^46^,^47^ Here, we found that hepatic total ceramide levels were significantly reduced in the group of mice supplemented with ω-3 PUFA-rich oil only compared with the HF control group (Figure S6A), with no effect in *fat-1* transgenic mice (Figure S5A). Numerous animal studies have reported that ω-3 PUFAs counter HF diet induced hepatic ceramide lipotoxicity thereby reducing inflammation, specifically TNF-α levels.^48^ Regarding the hepatic inflammation status, the endogenous production of ω-3 PUFA in tissues led to a global genotype effect on hepatic inflammation. Indeed, IL-2, IL-10 and, surprisingly, TNF-α levels were decreased in both LF- and HF diet-fed Fat-1 mice (Figure S5B). On the contrary, in the dietary model, ω3-PUFA supplementation also induced a significant decrease of hepatic IL-2, IL-3, and IL-10 levels compared with control (Figure S6B), but while we observed a significant lower ceramide level, unfortunately this did not translate into significantly lower TNF-α levels (Figure S6B).

While both models exhibited a reduction in the ω-6/ω-3 ratio, ω-3 PUFA supplementation led to a greater improvement of hepatic steatosis and inflammatory lipid mediator profile, compared with endogenous production in Fat-1 mice. Interestingly, only diet (LF or HF) modified the hepatic bile acid profile, whereas no effect of either endogenous or exogenous ω-3 PUFA was observed (Table S4).

### The Endogenous Production and Supplementation of Ω-3 PUFA Differentially Affect the Hepatic and Skeletal Muscle Endocannabinoidome

**Figure 5.**
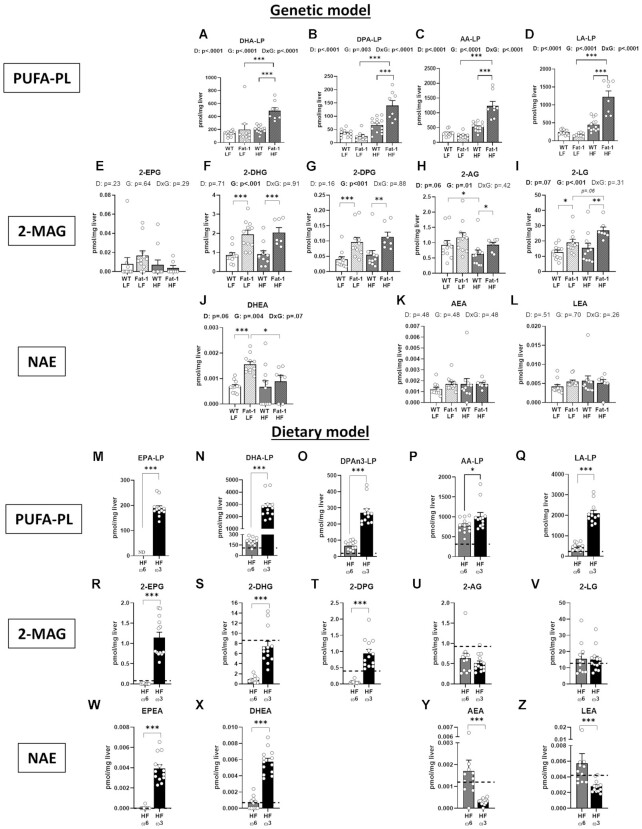
The hepatic endocannabinoidome is differentially impacted by the endogenous production and supplementation of ω-3 PUFA. Panels (**A–L**) refer to the genetic model and (**M**–**Z**) to the dietary model. (**A, N**) docosahexaenoic (DHA), (**M**) eicosapentaenoic (EPA), and (**B, O**) docosapentaenoic (DPA) ω-3 PUFAs. (**C, P**) Arachidonic (AA), (**D, Q**) linoleic acid (LA) ω-6 PUFAs. 2-monoacylglycerol (2-MAG) form of (**E, R**) EPA, (**F, S**) DHA, (**G, T**) DPA, (**H, U**) AA, and (**I, V**) LA PUFA precursors. *N*-acylethanolamine (NAE) form of (**W**) EPA, (**J, X**) DHA, (**K, Y**) AA, and (**L, Z**) LA PUFA precursors. Values are means ± SEM of *n* = 9–13 mice per group. For the genetic model: two-way ANOVA followed by Tukey’s post-hoc test. *P*-values of main effects for diet (D) and gene (G) or diet × gene (D × G) interaction are recorded under the title of each graph. For the dietary model: one-way ANOVA followed by Dunnett’s post-hoc test compared to HF-ω6 group. Dotted line represents LF-ω6 group as a reference. *P < .05, **P < .01, ***P < .001.

Endocannabinoids and their related bioactive lipids are ultimately synthesized from the corresponding PUFA-containing phospholipids (and in the case of 2-MAGs, also from PUFA-containing-lysophospholipids) and their levels can be influenced by dietary FA.^7^,^8^ Somewhat surprisingly lysophospholipid (LP)-EPA was not detectable and/or quantifiable in either WT or *fat-1* mice regardless of diet. As expected both DHA-LP and DPA-LP (Figure 5A and B) were significantly increased by endogenous production of ω-3 PUFA. Increased ω-3 PUFA tissue concentrations did not result in reduced ω-6 related AA or LA concentrations (Figure 5C and D).

Thus, we investigated the impact of ω-3 PUFA supplementation or endogenous production on hepatic endocannabinoids and related NAEs and 2-MAGs and their FA precursors. The EPA-derived lipid mediators *N*-eicosapentaenoyl-ethanolamine (EPEA) was not detected in the liver and while 2-eicosapentaenoyl-glycerol (2-EPG) levels were not different between groups (Figure 5E). This is consistent with the reported lack of detection of EPA. However, and as expected, Fat-1 mice displayed higher levels of 2-MAG derivatives of DHA and DPA (2-DPG and 2-AG, *P* < .001 and *P* < .01, respectively), but unexpectedly, also those of AA and LA (2-AG and 2-LG, *P* < .05 and *P* < .01, respectively) compared with their WT counterparts, regardless of diet treatments (Figure 5F–I). Fat-1-LF mice showed an increase in hepatic *N*-docosahexaenoyl-ethanolamine (DHEA) concentration compared with WT-LF mice (*P* < .001), but this effect was abrogated with the HF diet (Figure 5J). However, the NAE derivatives of the ω-6 PUFA, AEA and LEA, were not modified by either diet or genetic model (Figure 5K and L). Without significant impact on hepatic palmitic acid concentration (*P* = .07), palmitic acid-containing MAGs and NAEs were surprisingly increased in transgenic mice where the effect on *N*-palmitoyl-ethanolamine (PEA) was observed in Fat-1-LF mice (*P* < .001) but not in their HF-fed counterparts (Figure S7A–C). We also observed a decrease of OA in transgenic animals and a counterintuitive increase of 2-oleoyl-glycerol (2-OG) in Fat-1-HF mice (*P* < .01) as well as an increase of *N*-oleoyl-ethanolamine (OEA) in both groups of Fat-1 mice (genetic effect, *P* = .02) (Figure S7D-F). HF-ω3 mice also displayed higher levels of the corresponding 2-MAGs containing ω-3 PUFA—ie, 2-EPG, 2-DHG and 2-DPG (*P* < .001)—while no difference was observed in the levels of AA and LA-containing 2-MAGs (Figure 5R–V).

Unlike the Fat-1-HF, the HF-ω3 had a significant increase in EPA-LP levels (Figure 5M) as well as significantly higher levels of DHA-LP and DPA-LP (Figure 5N and O) like the genetic model. The elevated LP levels were reflected in the ω3 2-MAG and NAE concentrations. Both ω6 PUFA, AA-LP, and LA-LP, were significantly increased with ω3 supplementation (Figure 5P and Q) but this did not result in an increase in related 2-MAG molecules (Figure 5U and V). Contrary to the genetic model, NAE levels were consistent with those of 2-MAGs as we reported an increase of EPEA and DHEA (*P* < .001), and a decrease in AEA and LEA (*P* < .001) in HF-ω3 mice compared with HF-ω6 mice (Figure 5W–Z). Lower hepatic oleic and palmitic acid levels were observed in HF-ω3 compared with HF-ω6 mice, but no effect was detected in 2-MAGs and NAEs containing these FA (Figure S7G–M). Thus, the hepatic concentrations of ω-3 2-MAGs and NAEs similarly increased in both models, with a greater effect in the dietary model. However, the two models differentially affected the hepatic concentrations of non-ω3 2-MAGs and NAEs, since PEA and/or OEA and/or 2-OG were sensitive only to the endogenous production of ω-3 PUFA, while ω-6 NAEs (but not 2-MAGs) were only decreased by ω-3 PUFA administration.

The muscle endocannabinoidome profile was quite similar to that of liver, but with some exceptions (Figures S7 and S8). In the gastrocnemius, we detected a genotype effect and an increase of 2-EPG and DHEA concentrations in Fat-1 mice (*P* < .001) (Figure S8A–E). The genotype effect observed in the liver for 2-LG (*P* = .03) and 2-AG (*P* = .002) was only driven by the WT-LF group in the muscle (Figure S8F and H), and we did not find any increase in 2-OG and OEA levels in Fat-1-HF mice in this tissue (Figure S8L and M). In the dietary model, ω-3 PUFA administration decreased gastrocnemius concentration of 2-LG (*P* < .01) and 2-AG (*P* < .001) (Figure S8S and U).

Finally, we measured the hepatic expression of *Cnr1*, coding for the cannabinoid receptor type-1 (CB_1_), which is involved in the regulation of feeding, energy expenditure and reward system, and hepatic lipogenesis and insulin sensitivity, and is activated by AEA and 2-AG, but we did not detect a significant difference in either genetic Fat-1 mice or the dietary model, indicating that in the current dietary and genetic conditions endocannabinoid signaling is driven by ligand concentration and not receptor quantity (Figure S7N–O).

### ω-3 PUFA-derived Endocannabinoidome Mediators Reduce Lipid Accumulation in Hepatocytes

**Figure 6.**
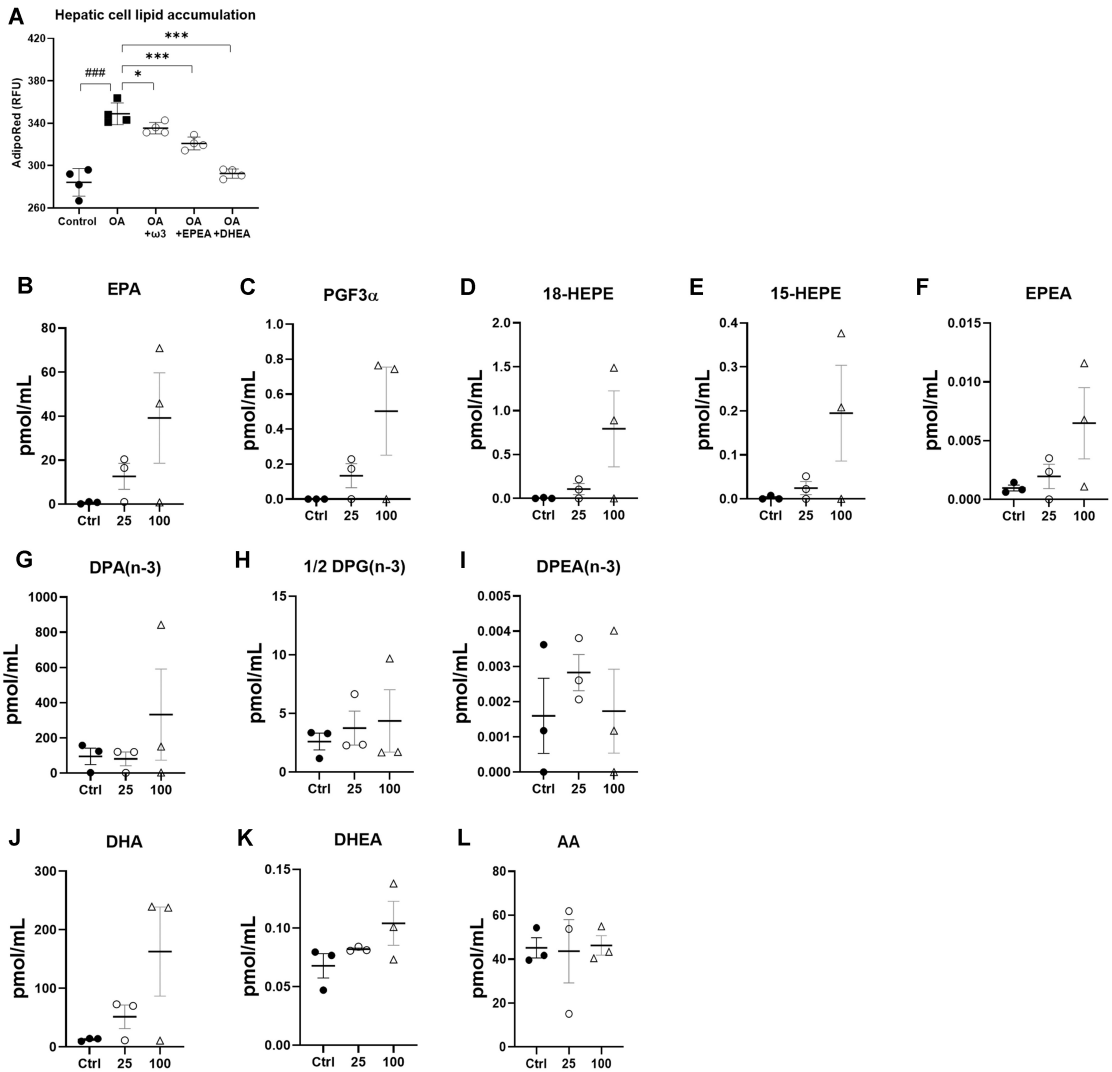
ω-3 PUFA-derived endocannabinoid-like mediators decrease lipid accumulation in hepatic cells. **(A)** HepG2 cells were treated with FBS media (Control), FBS media supplemented with 200 um oleic acid (OA) or the OA media + an EPA/DHA mix (Om3 2:1) or 50 nm*N*-eicosapentaenoyl-ethanolamine (EPEA) or 50 nm*N*-docosahexaenoyl-ethanolamine (DHEA) for 48 h. **(B–L)** HepG2 cells were treated with FBS media (Control), and 25 or 100 mmol of ω3-PUFA (EPA/DHA 2:1 mix) for 24 h.Values are means ± SEM of *n* = 4 performed in triplicate. Oleic acid group was compared to Control through a two-tailed *t*-test: ^###^*P *< .001. A one-way ANOVA followed by Dunnett’s post-hoc test was performed to determine the significant effect of ω3-PUFA and their NAEs derivatives:^*^P < .05 ***P < .001.

To understand better, the role of ω-3 PUFA-containing NAEs in liver steatosis prevention in the dietary model, we further performed in vitro experiments. We first observed that HepG2 cells exposed to OA during 24 h significantly increased lipid accumulation compared to control (Figure 6A). While co-treatment of OA-treated hepatic cells with the combination of the two ω-3 PUFA significantly decreased lipid accumulation, diminished lipid accumulation was significantly greater with either EPEA or DHEA single co-administration, with the level of lipid accumulation similar to that of control cells (no OA) for DHEA (Figure 6A). This finding is in agreement with the observed reduced lipid accumulation in the dietary model of ω-3 PUFA treatment, where DHEA and EPEA were significantly increased and hepatic TG was significantly decreased (Figure 4V), whereas in the genetic model, there was no significant increase in either EPEA or DHEA and no significant improvement in hepatic TG (Figure 4H). Therefore, this finding suggests that the two NAEs are very likely responsible for the lipid-lowering effects of exogenous ω3-PUFA in the liver. Accordingly, when hepatocytes were treated with ω-3 PUFA under the same conditions used to induce lipid accumulation, a trend towards increased levels of DHA and, particularly, EPA metabolites, including EPEA, was observed (Figure 6B–L).

### Multiple Factor Analysis Highlights the Role of TNF-α in ω3-PUFA-driven Effects

**Figure 7.**
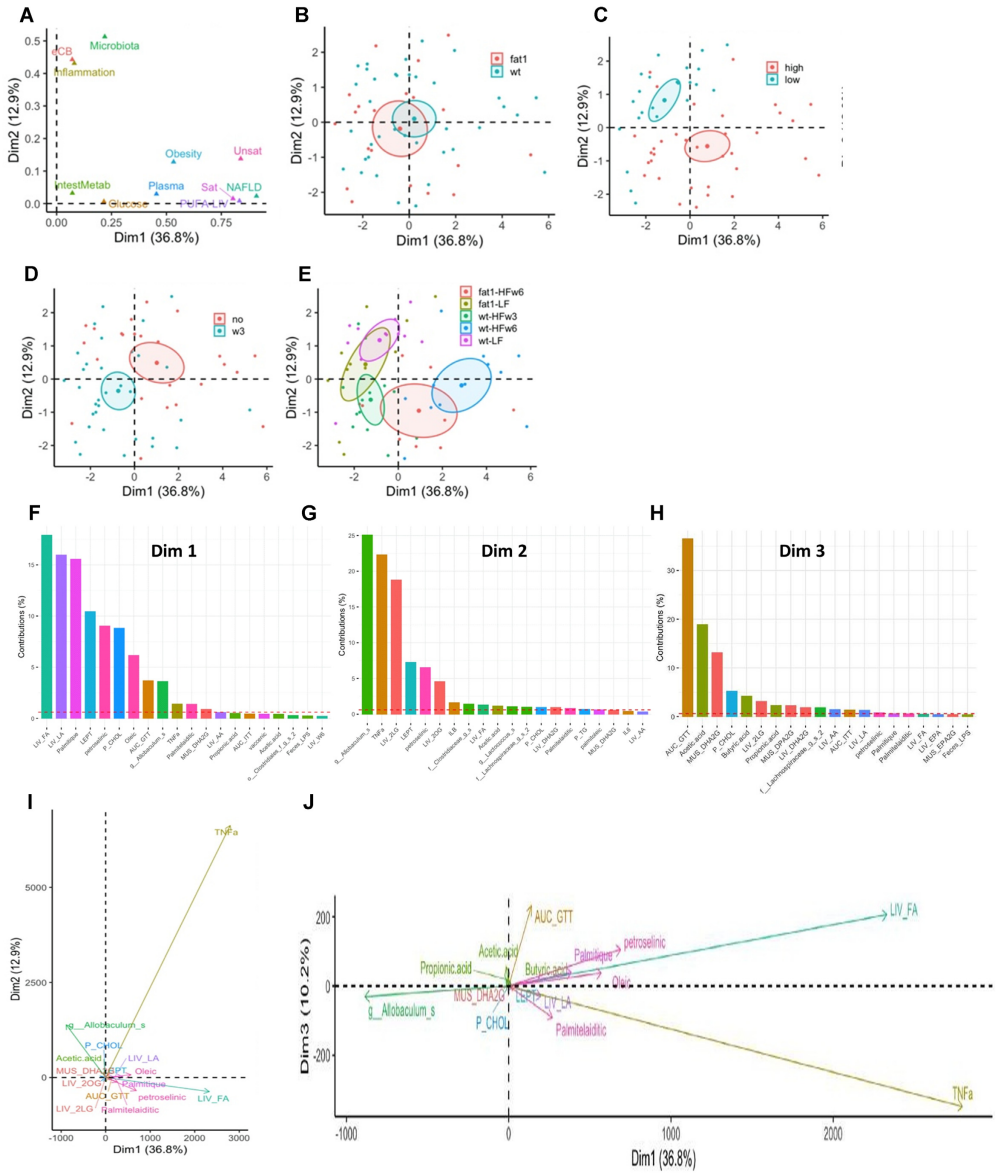
Multiple factor analysis (MFA) highlights the importance of the gut-liver axis in ω-3 effects. (**A**) Loading plot represent the influence of the top 11 variables with the highest contribution. (**B–E**) Ellipses show the effect of (**B**) genetic (*fat*1 vs WT), (**C**) dietary lipid content (low vs high), (**D**) ω-3 PUFA intake, and (**E**) combined conditions on mice. *P*-values reported on the bottom right of each MFA were obtained by permutational multivariate analysis of variance (PERMANOVA). Barplots representing the contribution of the top 20 variables to the MFA plot axis for (**F**) dimension 1 and (**G**) dimension 2. (**H**) Loading plot represents the influence of all the variable groups on the MFA plot on dimensions 1 and 2.

To better understand the role of ω-3 PUFA in metabolism, we next performed an MFA. Quantitative variables (*n* = 165) were grouped into 11 factors (Figure 7A, Table S5) representing various metabolic pathways or biological features (eg, gut microbiota populations, hepatic FA, body composition) allowing us to determine each factor’s contribution to the observed differences between ω-3 PUFA enrichment models and to evaluate overall changes between mice. Mice tended to differ based on their genetic background (WT vs Fat-1, *P* = .08, Figure 7B) and were significantly differentiated by dietary fat content (high vs low, *P* = .002, Figure 7C) or the presence/absence of ω-3 PUFA, regardless of dietary fat content (*P* = .002, Figure 7D). Clusters corresponding to each HF diet treatment group position at similar levels on the dimension 2 axis (Figure 8E), while the LF diet mice were at a similar level as the WT-HF ω3 mice on dimension 1 axis. The mice fed a LF diet (WT or Fat1) had a similar profile for the variables contributing to dimension-1 axis along with the HF-ω3 mice. More specifically, the genera *Allobaculum* (from the factor Microbiota Figure 7A) was associated with the LF- diet (dimension-2, Figure 7G) and HF-ω3 (dimension-1, Figure 7F) mice while the liver FA species were associated with the WT-HF-ω6 mice (Figure 7H).

The hepatic concentration of the proinflammatory cytokine TNF-α strongly differentiated the three HF diet-fed groups along dimension 2 (Figure 7G and H) and to a lesser degree dimension 1 (Figure 7F). When analyzed within each treatment model the reduction in hepatic TNF-α was not statistically significant (Figures S5 and S6) but was consistently reduced by the presence of ω-3 PUFA either genetically or through dietary means. Other liver cytokines (IL-1β and IL-6, Figures S5 and S6) contributed to differentiating the groups but to a much lesser extent.

The strongest individual variable that contributed to the differences along axis 1 (ω-3 vs ω-6 PUFA, Figure 7E) is total hepatic FA concentration, specifically hepatic LA and palmitic acid (LIV_FA, LIV_LA, and Palmitic) and plasma leptin (Figure 7F). This was supported by the FA profiles (Table S1) and reduced body weight gain and increased plasma leptin (Figure 1B, I, and N) for the LF and ω-3 PUFA groups (Fat-1 or WT-ω3). The factors Sat (total hepatic saturated FA content), Unsat (total hepatic monounsaturated FA content) and NAFLD cluster along dimension 1 and while there is a less pronounced difference between the dietary and endogenous ω-3 models, the MFA in conjunction with the individual FA analysis (Table S1) provides insight into the observed difference in hepatic steatosis between these two groups (Figure 4F and T). From individual hepatic FA concentrations, we observed that dietary ω-3 PUFA significantly reduced total hepatic saturated FA content and specifically palmitic, eicosanoic, and stearic but there was no genetic effect (Fat-1, *P* = .07). Vaccenic acid, OA, and overall monounsaturated FA were reduced with dietary ω-3 PUFA, while the Fat-1 genotype did not have this effect (*P* = .05) and finally while the ω-6/ω-3 PUFA ratio (a variable within the NAFLD factor) was significantly lower in both ω-3 PUFA models, dietary ω-3 PUFA had a greater effect (Table S1). Therefore, our results are in line with our hypothesis that the supplementation of ω-3 PUFA has a more profound influence on the hepatic FA status, with the subsequent increased production of EPEA and DHEA at the expense of endocannabinoids possibly resulting in a lower hepatic fat accumulation.

## Discussion

Our work compared the impact of endogenous ω-3 PUFA production, through *fat-1* expression, vs exogenous ω-3 PUFA by fish oil consumption, on HF-induced metabolic dysfunctions in the context of NAFLD development. We thus focused on the gut-liver axis, and also determined how both models modulated the gut microbiota composition and the hepatic endocannabinoidome (see Table 1). One limitation of comparing outcomes between the two methods of ω-3 PUFA delivery was that the genetic model attained lower levels of tissue ω-3 PUFA. However, we have confirmed using MFA that ω-3 content did not significantly differentiate the experimental groups. The genetic model had lower ω-3 PUFA tissue content leading to a higher hepatic ω-6/ω-3 PUFA ratio compared with the dietary model but nonetheless significantly lower than that of the WT-HF group. We first posit that this was primarily due to the use of hemizygous *fat-*1 mice, which we used to obtain an ω-6/ω-3 PUFA ratio in HF-fed mice comparable to chow-fed mice, as previously observed.^23^ Studies investigating the effects of ω-3 PUFA on NAFLD have used both hetero- and homozygous *fat-1* mice with various concentrations of liver PUFA obtained.^49–51^ Heterozygous *fat-1*animals relying solely on a high-fat diet to induce NAFLD^50^,^51^ achieved lower ω-6/ω-3 ratios and improvement in NAFLD compared to streptozotocin-induced NAFLD in homozygous *fat-1* mice (1.5- to 2-fold, respectively).^49^ It thus appears that background diet, specifically percentage and type of fat, plays a more important role than the genetic *fat-1* model used to modulate the hepatic ω-6/ω-3 ratio. Therefore, using homozygous animals would not have guaranteed similar concentrations of ω-3 fatty acids as the ω-3 supplemented animals in this study.

**Table 1.** Summary of genetic and dietary effects on metabolism

	**Dietary Model**	**Genetic Model**	**Comments**
**Body composition**	↘body weight gain ↘ energy efficiency of diet ↘ liver weight	↘ body weight gain ↘ energy efficiency of diet ↘ liver weight in HF-fed mice	Fat-1 model tends to decrease body weight gain induced by HF diet
**Insulin and glucose tolerance tests**		↗ insulin sensibility and glucose tolerance	Supplementation has no effect during ipITT and oGTT
**Plasma biomolecules**	↘ leptin ↘ TG and cholesterol	↘ cholesterol in HF-fed mice	
**Hepatic lipids and their derivatives**	↘ TG, total FA, palmitic acid, OA, and ω-6/ω-3 PUFA ratio ↘ Total ω-6 PUFA, AA, and LA ↗ Total ω-3 PUFA, EPA, DHA, and DPA ↗ 17-HDHA and 18-HEPE ↘ LTB_4_	↘ Total FA in HF-fed mice and ω-6/ω-3 PUFA ratio↘ Total ω-6 PUFA and LA in HF-fed mice ↗ Total ω-3 PUFA, DHA, DPA in HF-fed mice	Supplementation of ω-3 PUFA markedly improves hepatic steatosisω-6/ω-3 PUFA ratio:HF-ω3 vs HF-Fat1:13.9 vs 1.7
	↗ 2-MAGs: 2-EPG, 2-DHG, 2-DPGNAEs: ↗ EPEA, DHEA and ↘ AEA, LEA	↗ 2-MAGs: 2-DHG, 2-DPG, 2-AG, 2-LG ↗ 2-OG in HF-fed mice	
**Hepatic bile acids (BA)**	pM/mg: ↗ CA, GCA%: ↗ CA,↘ TCDCA, TUDCA	Trend for ↗ total primary BA, total BA, and total conjugated BA	Few effects on hepatic BA profile in both models
**Hepatic cytokines**	↘ IL-2, IL-3, IL-10	↘ IL-1β, IL-2, IL-6, IL-10, INF-γ, RANTES, TNF-α	
**Gut microbiota composition (feces)**	↘ *Lachnospiraceae* and *Ruminococcaceae* families, *Parabacteroides, Dorea, Proteus, Coprococcus* genera ↗ *Turicibacter* and *Allobaculum* genera	↘ S24-7, *Parabacteroides, Oscillospira* genera ↗ *Erysipelotrichaceae* family	Similar gut microbiota profiles between both models *Allobaculum* genus belongs to the *Erysipelotrichaceae* family
**Caecal SCFA**	↗ Caecum weight ↗ propionate, isovalerate, isobutyrate		
**Skeletal muscle lipid mediators**	↗ 17-HDHA, 18-HEPE		
**Gastrocnemius endocannabinoids**	↗ 2-EPG, EPEA, E-DPG, 2-DHG, DHEA ↘ 2-LG, LEA, 2-AG, AEA	↗ 2-EPG, EPEA, 2-DPG, 2-DHG, DHEA	

Data were reduced into factors representing all variables to perform MFA, which allowed us to identify shared but also model-specific observations between the genetic and supplemented models, highlighting the key role of the gut microbiota and inflammatory cytokines in metabolic health. The strongest distinguishing feature was the proinflammatory cytokine TNF-α. Reducing inflammation and resolving inflammation are both features of the ω-3 PUFA supplementation or endogenous production models. A reduction in inflammation can accompany a decrease in the ω-6/ω-3 PUFA ratio and lower saturated fat tissue concentrations, both of which occurred in the liver by Fat-1 driven transgenic production of ω-3 PUFA, and to a greater extent by daily ω-3 PUFA intake. This reduction in tissue inflammation and TNFα may be linked to decreased activation of the of NF-κB pathway.^52^,^53^

Lipidomic analyses examined the relationship between lipid species and NAFLD with the primary intention of identifying biomarkers for earlier detection of fatty liver.^54^,^55^ Although numerous lipid signatures differentiating liver lipid accumulation stages, have been identified,^56^ the majority of species are sphingolipids and phospholipids.^54^,^57^ Plasmalogens, ether-linked phospholipids, are vital to membrane integrity, which is favorable to secondary messengers and cell signal transduction.^58^ Although not normally abundant, a significant reduction in hepatic plasmalogen concentrations was reported in patients with NASH,^59^ and with Zellweger syndrome characterized by hepatic dysfunction and fatty liver.^60^ Both methods of increased ω-3 PUFA production significantly increased the total ether-linked PC and PE concentrations but only exogenous ω-3 PUFA supplementation resulted in improved hepatic steatosis status. Hepatic PC, is made from choline via the CDP-choline pathway and by PE N-methyltransferase (PEMT).^58^ Mice lacking *Pemt* exhibited liver dysfunction similar to NASH when fed a choline deficient diet where the PC:PE ratio decreases by >50%, leading to loss of membrane integrity and increased presence of hepatocyte ballooning and inflammatory response.^61^ Ling et al. showed that a decreased hepatic PC/PE ratio is a predictor of NAFLD and survival following partial hepatectomy. Hepatic PC/PE is therefore inversely correlated with the development of steatosis and inflammation in the progression of NAFLD.^45^ In the current study the PC:PE ratio was a better predictor of hepatic steatosis status as only the exogenous ω-3 PUFA supplementation increased this ratio. These PUFAs can be cleaved and metabolized into potent second messenger molecules, such as protectins and resolvins, to induce anti-inflammatory and anti-apoptotic effects.^62^ Previously, we have reported an improvement in insulin sensitivity with increased endogenous mediator concentration^23^ but this is the first time we have reported hepatic levels of ω-3 PUFA derived mediators by exogenous supplementation. We found that exogenous supplementation significantly increases both 17-HDHA. 18-HEPE and PDX in association with improved inflammation and steatosis status but not improved insulin sensitivity.

Possibly independently of their role as hepatic biomarkers, phospholipids, plasmalogens, and lysophosholipids, play a crucial role as biosynthetic precursors of endocannabinoids and related mediators. Previous preclinical and clinical studies clearly demonstrated that the endocannabinoid system plays a pivotal role in energy metabolism homeostasis. It has been reported that tissue and circulating concentrations of some endocannabinoid species derived from the ω-6 PUFA AA—such as AEA and 2-AG—are increased, while ω-3 PUFA-containing NAEs and MAGs are decreased, under pathophysiological conditions.^63^ In the present study, both models of ω-3 PUFA-tissue enrichment significantly modulated the liver endocannabinoidome compared to HF diet alone, rich in precursors for ω-6 PUFA. In particular, both endogenous production and ω-3 PUFA supplementation increased 2-DHG and DHEA deriving from DHA, and 2-DPG deriving from DPA. However, ω-3 PUFA supplementation induced a greater hepatic accumulation of these compounds and also increased the level of 2-EPG and EPEA deriving from EPA, compared with endogenous ω-3 PUFA production. Interestingly, further in-vitro analysis demonstrated that HepG2 cells treated with specific NAEs, EPEA, and DHEA, significantly reduced lipid accumulation compared to control, which corroborates the significant differences observed in hepatic lipid and endocannabinoid profiles between our two models. Additionally, ω-3 PUFA supplementation markedly decreased hepatic concentrations of proinflammatory mediators AEA and LEA as previously described,^8^,^10^,^11^,^13^,^17^,^18^ while endogenous ω-3 PUFA production had no statistically significant effect. This last result may be explained by the fact we used hemizygous *fat-1^+/^^−^* mice, while in a previous study, the authors reported that hepatic AEA levels are decreased in homozygous *fat-1^+/+^* mice.^13^ However, it must be pointed out that, regardless of the approach used to induce ω-3 PUFA-tissue enrichment, we found a greater and more general impact on the hepatic levels of ω-3 PUFA-containing endocannabinoidome mediators, rather than on AEA, 2-AG and their non-ω-3 PUFA-derived congeners.

It is important to note that AEA was previously described as a “gate opener” as diet-induced obesity in rodents enhanced liver AEA levels^64^,^65^ and its increase in the intestine was related to higher gut permeability, circulating endotoxemia, and inflammation.^19^ It has also been reported that high hepatic AEA levels inhibit hepatic insulin sensitivity, and cause hepatic TG accumulation and steatosis via cannabinoid CB_1_ receptor activation.^64^,^65^ Contrary to our studies, Bidu et al.^66^ and Kaliannan et al.^67^ reported an improvement of hepatic steatosis in homozygous *fat-1*^+/+^ mice. In contrast, a recent paper elegantly demonstrated that the deletion of *Cnr1* did not protect from obesogenic diet-induced NAFLD in both in-vivo and in-vitro experiments^68^ raising controversy on the mechanisms involved in liver steatosis, and urging for further studies. Here, we did not observe marked HF diet-induced changes in MAG and NAE levels compared with the LF diet-fed mice. This was also reported in Lacroix et al.^21^ who, however, could detect some changes in the plasma. This apparent discrepancy with the literature^21^,^69^ may be explained by differences in control diets used between protocols, but it could also be related to differences in the gut microbiota composition observed in different animal facilities. In addition, further analysis performed here failed to show any differences in *Cnr1* expression between groups, which is consistent with the findings of Wang et al.^68^

Despite the different effects caused on fatty liver, the two models used here showed a similar protection against HF-induced hepatic pro-inflammatory cytokine production. This effect might be afforded by an even small (such as that observed in *fat-1^+/^^−^* mice) elevation of the hepatic levels of ω-3 PUFA-containing endocannabinoidome mediators, particularly since these compounds have been widely described to have potent anti-inflammatory effects,^16^ possibly via activation of multiple anti-inflammatory receptors, including GPR110 and PPARγ for DHEA^70^,^71^ and CB_2_ for 2-EPG, DHEA, DPEA, and EPEA.^72^,^73^ Indeed, CB_2_ receptors have been suggested as a potential therapeutic target for steatohepatitis and NAFLD^74^ and are recognized as anti-inflammatory receptors in mice.^75^ Further studies will need to investigate this hypothesis.

Our two models also similarly affected the gut microbiota composition, which is consistent with the literature. Separate studies reported that *fat-1* mediated ω-3 PUFA production^5^,^66^ as well as ω-3 PUFA supplementation^76^,^77^ are able to modulate bacterial populations. Here, stronger modulations were observed at the family/genus levels after 12-wk ω-3 PUFA supplementation compared with the endogenous ω-3 PUFA production. One of the most striking changes was the important and similar increase in the relative abundance of *Allobaculum* (from *Erysipelotrichales* order) in feces of both HF-ω3 and HF-Fat-1 mice, which exhibit a healthier phenotype compared with their HF control. This is of major interest given that the presence of *Allobaculum* has been repeatedly reported to be decreased following HF diets and negatively correlated with weight gain, type 2 diabetes, and NAFLD.^78–81^ Previous studies also reported a negative association between *Allobaculum* and cholesterol metabolism,^82^,^83^ which is consistent with our results since we found a decrease in plasma cholesterol with both models.

Interestingly, adipose tissue-specific *N*-acylphosphatidylethanolamine phospholipase D (NAPE-PLD) knockout (KO) mice display an obese phenotype and dyslipidemia, which have been linked to a shift in gut microbiota populations and a decrease in particular of the *Allobaculum* genus.^84^*N*-acylphosphatidylethanolamine phospholipase D is a key enzyme involved in the biosynthesis of NAEs, and, accordingly, these KO mice exhibit lower adipose tissue levels of PEA and OEA. These NAEs were found here to be increased in the liver of HF-Fat-1, but not of HF-ω3 mice, and this may suggest that the modulation of peripheral levels of these anti-inflammatory mediators and PPARα ligands^85^ might be related to changes in *Allobaculum* sp. relative abundance, and compensate for the reduced potential anti-inflammatory effect due the lower increase of ω-3 PUFA-derived endocannabinoidome mediators observed in HF-Fat-1 as compared to HF-ω3 mice. Recently, hepatocyte-specific NAPE-PLD KO mice were also developed and found to exhibit HF-induced steatosis and liver inflammation concomitantly with lower hepatic OEA, LEA, and DHEA levels.^86^ Unfortunately, the composition of the gut microbiota of these mice was not reported.

The increased release of propionate, a SCFA produced by some commensal bacteria following fiber fermentation, into the colon is associated with a decrease of intrahepatocellular lipid accumulation in adult subjects with NAFLD.^87^ The comparable level of protection against HF-induced hepatic inflammation observed with both models of ω-3 PUFA production maybe attributable to increased *Allobaculum* abundance. Short chain fatty acids, produced by *Allobaculum*, are associated with decreased gut inflammation resulting from colitis^88^ or alcohol-induced liver damage^89^,^90^ suggesting that *Allobaculum* may contribute to an anti-inflammatory phenotype. *Allobaculum* has also shown to be negatively correlated with ALT, AST, and TNFα resulting in a reduced hepatic steatosis suggesting protection against inflammation and oxidative stress through SCFA.^90^ In the current study, an increase of propionate concentration in caecal content was only found in HF-ω3 mice, in which hepatic steatosis was totally inhibited. Propionate has also been related to hypocholesterolemic effects,^91^,^92^ which could partly explain why HF-ω3 mice exhibited lower plasma cholesterol levels than Fat-1-HF mice. Since species that belong to *Allobaculum* are butyrate but not propionate producers, the latter SCFA could be issued from bacterial cross-feeding. Indeed, previous studies showed that bacteria-originated SCFA (ie, butyrate) become subsequently available as nutrients for other populations, leading to their growth and metabolite production (ie, propionate).^93^,^94^ A recent study also highlighted the differences in gut microbiota between caecal content and feces, showing that the *Allobaculum* genus was less represented in the caecum (9%) compared to the colon (22%).^95^ These results could explain our lack of a significant increase of butyrate concentrations in caecal content despite the high proportions of *Allobaculum* sp. in the fecal samples from both models.

The different ω-6/ω-3 PUFA ratios between the two models, while possibly explaining why the tissue concentrations of ω-6 and ω-3 PUFA-derived endocannabinoidome mediators were modified in a manner qualitatively, but not quantitatively, similar, allowed us to speculate on which of the differential metabolic changes observed might have been (1) due to, (2) sensitive to even small, or (3) independent from, alterations in endocannabinoidome signaling. This allowed us to identify other potential factors, such as TNFα and gut microbiota changes, which may represent common mechanisms for the metabolic ameliorations observed in the two models. It is important to remember that the observed tissue concentrations of mediators such as 2-MAGs and NAEs does not necessarily reflect cellular receptor site concentrations, where these molecules, due to their lipophilic nature, might achieve much higher local concentrations than those detected in whole tissues.

In conclusion, this comparative study revealed that the gut-liver axis plays an important role in the beneficial effects of ω-3 PUFAs supplementation on NAFLD prevention. On the other hand, the whole body-tissue enrichment in ω-3 PUFAs induced in the Fat-1 model ameliorated global metabolic homeostasis, as shown by its preventive impact on insulin sensitivity and glucose tolerance. This suggests that several metabolic tissues are involved in this tight balance, and not only the liver. The common effects reported in both models might be partly explained by a potential interplay between gut microbiota populations, particularly *Allobaculum*, while the endocannabinoidome through the emerging class of ω-3 PUFA-derived endocannabinoid-like lipid mediators, might be more strongly involved in reduced liver fat accumulation.

## Supplementary Material

zqac069_Supplemental_File

## Data Availability

The datasets generated during the current study are available from the corresponding author on reasonable request.

## References

[1] Leung C , Rivera L, Furness JB, Angus PW. The role of the gut microbiota in NAFLD. Nat Rev Gastroenterol Hepatol. 2016;13(7):412–425. 27273168 10.1038/nrgastro.2016.85

[2] Machado M , Cortez-Pinto H. Diet, microbiota, obesity, and NAFLD: a dangerous quartet. Int J Mol Sci. 2016;17(4):481. 27043550 10.3390/ijms17040481 PMC4848937

[3] Miele L , Marrone G, Lauritano C et al. Gut-liver axis and microbiota in NAFLD: insight pathophysiology for novel therapeutic target. Curr Pharm Des. 2013;19(29):5314–5324. 23432669

[4] Costantini L , Molinari R, Farinon B, Merendino N. Impact of omega-3 fatty acids on the gut microbiota. Int J Mol Sci. 2017;18(12):2645. 29215589 10.3390/ijms18122645 PMC5751248

[5] Kaliannan K , Wang B, Li X-Y, Kim K-J, Kang JX. A host-microbiome interaction mediates the opposing effects of omega-6 and omega-3 fatty acids on metabolic endotoxemia. Sci Rep. 2015;5(1):11276. 26062993 10.1038/srep11276 PMC4650612

[6] Veilleux A , Di Marzo V, Silvestri C. The expanded endocannabinoid system/endocannabinoidome as a potential target for treating diabetes mellitus. Curr Diab Rep. 2019;19(11):117. 31686231 10.1007/s11892-019-1248-9

[7] Di Marzo V , Silvestri C. Lifestyle and metabolic syndrome: contribution of the endocannabinoidome. Nutrients. 2019;11(8):1956. 31434293 10.3390/nu11081956 PMC6722643

[8] Castonguay-Paradis S , Lacroix S, Rochefort G et al. Dietary fatty acid intake and gut microbiota determine circulating endocannabinoidome signaling beyond the effect of body fat. Sci Rep. 2020;10(1):15975. 32994521 10.1038/s41598-020-72861-3 PMC7524791

[9] Matias I , Cristino L, Di Marzo V. Endocannabinoids: some like it fat (and sweet too). J Neuroendocrinol. 2008;20(s1):100–109. 18426508 10.1111/j.1365-2826.2008.01678.x

[10] Batetta B , Griinari M, Carta G et al. Endocannabinoids may mediate the ability of (n-3) fatty acids to reduce ectopic fat and inflammatory mediators in obese Zucker rats. J Nutr. 2009;139(8):1495–1501. 19549757 10.3945/jn.109.104844

[11] Piscitelli F , Carta G, Bisogno T et al. Effect of dietary krill oil supplementation on the endocannabinoidome of metabolically relevant tissues from high-fat-fed mice. Nutr Metab (Lond). 2011;8(1):51. 21749725 10.1186/1743-7075-8-51 PMC3154144

[12] Berge K , Piscitelli F, Hoem N, Silvestri C, Meyer I, Banni S, Di Marzo V. Chronic treatment with krill powder reduces plasma triglyceride and anandamide levels in mildly obese men. Lipids Health Dis. 2013;12(1):78. 23706001 10.1186/1476-511X-12-78 PMC3680309

[13] Demizieux L , Piscitelli F, Troy-Fioramonti S et al. Early low-fat diet enriched with linolenic acid reduces liver endocannabinoid tone and improves late glycemic control after a high-fat diet challenge in mice. Diabetes. 2016;65(7):1824–1837. 27207550 10.2337/db15-1279

[14] Freitas HR , Isaac AR, Malcher-Lopes R, Diaz BL, Trevenzoli IH, De Melo Reis RA. Polyunsaturated fatty acids and endocannabinoids in health and disease. Nutr Neurosci. 2018;21(10):695–714. 28686542 10.1080/1028415X.2017.1347373

[15] Watson JE , Kim JS, Das A. Emerging class of omega-3 fatty acid endocannabinoids & their derivatives. Prostaglandins Other Lipid Mediat. 2019;143:106337. 31085370 10.1016/j.prostaglandins.2019.106337 PMC6685292

[16] De Bus I , Witkamp R, Zuilhof H, Albada B, Balvers M. The role of n-3 PUFA-derived fatty acid derivatives and their oxygenated metabolites in the modulation of inflammation. Prostaglandins Other Lipid Mediat. 2019;144:106351. 31260750 10.1016/j.prostaglandins.2019.106351

[17] Rossmeisl M , Macek Jilkova Z, Kuda O et al. Metabolic effects of n-3 PUFA as phospholipids are superior to triglycerides in mice fed a high-fat diet: possible role of endocannabinoids. PLoS One. 2012;7(6):e38834. 22701720 10.1371/journal.pone.0038834 PMC3372498

[18] Rossmeisl M , Pavlisova J, Janovska P et al. Differential modulation of white adipose tissue endocannabinoid levels by n-3 fatty acids in obese mice and type 2 diabetic patients. Biochim Biophys Acta Mol Cell Biol Lipids. 2018;1863(7):712–725. 29626526 10.1016/j.bbalip.2018.03.011

[19] Cani PD , Plovier H, Van Hul M et al. Endocannabinoids—at the crossroads between the gut microbiota and host metabolism. Nat Rev Endocrinol. 2016;12(3):133–143. 26678807 10.1038/nrendo.2015.211

[20] Everard A , Plovier H, Rastelli M et al. Intestinal epithelial N-acylphosphatidylethanolamine phospholipase D links dietary fat to metabolic adaptations in obesity and steatosis. Nat Commun. 2019;10(1):457. 30692526 10.1038/s41467-018-08051-7 PMC6349942

[21] Lacroix S , Pechereau F, Leblanc N et al. Rapid and concomitant gut microbiota and endocannabinoidome response to diet-induced obesity in mice. mSystems. 2019;4(6):e00407–19. 31848310 10.1128/mSystems.00407-19 PMC6918026

[22] Kang JX , Wang J, Wu L, Kang ZB. Transgenic mice: fat-1 mice convert n-6 to n-3 fatty acids. Nature. 2004;427(6974):504. 14765186 10.1038/427504a

[23] White PJ , Arita M, Taguchi R, Kang JX, Marette A. Transgenic restoration of long-chain n-3 fatty acids in insulin target tissues improves resolution capacity and alleviates obesity-linked inflammation and insulin resistance in high-fat-fed mice. Diabetes. 2010;59(12):3066–3073. 20841610 10.2337/db10-0054 PMC2992767

[24] White PJ , Mitchell PL, Schwab M et al. Transgenic ω-3 PUFA enrichment alters morphology and gene expression profile in adipose tissue of obese mice: potential role for protectins. Metabolism. 2015;64(6):666–676. 25726444 10.1016/j.metabol.2015.01.017

[25] De Castro GS , Calder PC. Non-alcoholic fatty liver disease and its treatment with n-3 polyunsaturated fatty acids. Clin Nutr. 2018;37(1):37–55. 28139281 10.1016/j.clnu.2017.01.006

[26] Anhê FF , Roy D, Pilon G et al. A polyphenol-rich cranberry extract protects from diet-induced obesity, insulin resistance and intestinal inflammation in association with increased *Akkermansia* spp. population in the gut microbiota of mice. Gut. 2015;64(6):872–883. 25080446 10.1136/gutjnl-2014-307142

[27] Spahis S , Alvarez F, Dubois J, Ahmed N, Peretti N, Levy E. Plasma fatty acid composition in French–Canadian children with non-alcoholic fatty liver disease: effect of n-3 PUFA supplementation. Prostaglandins Leukot Essent Fatty Acids. 2015;99:25–34. 26066299 10.1016/j.plefa.2015.04.010

[28] Lamaziere A , Richard D, Barbe U et al. Differential distribution of DHA-phospholipids in rat brain after feeding: a lipidomic approach. Prostaglandins Leukot Essent Fatty Acids. 2011;84(1–2):7–11. 21109411 10.1016/j.plefa.2010.11.001

[29] Shillito B , Desurmont C, Barthélémy D et al. Lipidome variations of deep-sea vent shrimps according to acclimation pressure: a homeoviscous response?. Deep Sea Res.Part I Oceanogr Res Pap. 2020;161:103285.

[30] Folch J , Lees M, Stanley GHS. A simple method for the isolation and purification of total lipides from animal tissues. J Biol Chem. 1957;226(1):497–509. 13428781

[31] Mitchell PL , Nachbar R, Lachance D et al. Treatment with a novel agent combining docosahexaenoate and metformin increases protectin DX and IL-6 production in skeletal muscle and reduces insulin resistance in obese diabetic db/db mice. Diabetes Obes Metab. 2017;19(3):313–319. 27800648 10.1111/dom.12818

[32] Perazza LR et al. Fish oil replacement prevents, while docosahexaenoic acid-derived protectin DX mitigates end-stage-renal-disease in atherosclerotic diabetic mice. FASEB J Off Publ Fed Am Soc Exp Biol. 2021;35(5):e21559. 10.1096/fj.202100073R 33835594

[33] Barnett HY , Geys H, Jacobs T, Jaki T. Methods for non-compartmental pharmacokinetic analysis with observations below the limit of quantification. Stat Biopharm Res. 2021;13(1):59–70.

[34] García-Villalba R , Giménez-Bastida JA, García-Conesa MT, Tomás-Barberán FA, Carlos Espín J, Larrosa M. Alternative method for gas chromatography-mass spectrometry analysis of short-chain fatty acids in faecal samples. J Sep Sci. 2012;35:1906–1913. 22865755 10.1002/jssc.201101121

[35] Tomkovich S , Yang Ye, Winglee K et al. Locoregional effects of microbiota in a preclinical model of colon carcinogenesis. Cancer Res. 2017;77(10):2620–2632. 28416491 10.1158/0008-5472.CAN-16-3472 PMC5468752

[36] Edgar RC , Haas BJ, Clemente JC, Quince C, Knight R. UCHIME improves sensitivity and speed of chimera detection. Bioinformatics. 2011;27(16):2194–2200. 21700674 10.1093/bioinformatics/btr381 PMC3150044

[37] Edgar RC . Search and clustering orders of magnitude faster than BLAST. Bioinformatics. 2010;26(19):2460–2461. 20709691 10.1093/bioinformatics/btq461

[38] Desantis TZ , Hugenholtz P, Larsen N et al. Greengenes, a chimera-checked 16S rRNA gene database and workbench compatible with ARB. Appl Environ Microbiol. 2006;72(7):5069–5072. 16820507 10.1128/AEM.03006-05 PMC1489311

[39] Wang Q , Garrity GM, Tiedje JM, Cole JR. Naive Bayesian classifier for rapid assignment of rRNA sequences into the new bacterial taxonomy. Appl Environ Microbiol. 2007;73(16):5261–5267. 17586664 10.1128/AEM.00062-07 PMC1950982

[40] Bokulich NA , Subramanian S, Faith JJ et al. Quality-filtering vastly improves diversity estimates from Illumina amplicon sequencing. Nat Methods. 2013;10(1):57–59. 23202435 10.1038/nmeth.2276 PMC3531572

[41] Cole JR , Wang Q, Fish JA et al. Ribosomal Database Project: data and tools for high throughput rRNA analysis. Nucleic Acids Res. 2014;42(D1):D633–D642. 24288368 10.1093/nar/gkt1244 PMC3965039

[42] Chassaing B , Koren O, Carvalho FA, Ley RE, Gewirtz AT. AIEC pathobiont instigates chronic colitis in susceptible hosts by altering microbiota composition. Gut. 2014;63(7):1069–1080. 23896971 10.1136/gutjnl-2013-304909 PMC4089387

[43] Lagkouvardos I , Lesker TR, Hitch TCA et al. Sequence and cultivation study of *Muribaculaceae* reveals novel species, host preference, and functional potential of this yet undescribed family. Microbiome. 2019;7(1):28. 30782206 10.1186/s40168-019-0637-2 PMC6381624

[44] Paul S , Lancaster GI, Meikle PJ. Plasmalogens: a potential therapeutic target for neurodegenerative and cardiometabolic disease. Prog Lipid Res. 2019;74:186–195. 30974122 10.1016/j.plipres.2019.04.003

[45] Ling J , Chaba T, Zhu L-F, Jacobs RL, Vance DE. Hepatic ratio of phosphatidylcholine to phosphatidylethanolamine predicts survival after partial hepatectomy in mice. Hepatology. 2012;55(4):1094–1102. 22095799 10.1002/hep.24782

[46] Petersen MC , Shulman GI. Roles of diacylglycerols and ceramides in hepatic insulin resistance. Trends Pharmacol Sci. 2017;38(7):649–665. 28551355 10.1016/j.tips.2017.04.004 PMC5499157

[47] Summers SA , Chaurasia B, Holland WL. Metabolic messengers: ceramides. Nat Metab. 2019;1(11):1051–1058. 32694860 10.1038/s42255-019-0134-8 PMC7549391

[48] Walchuk C , Wang Y, Suh M. The impact of EPA and DHA on ceramide lipotoxicity in the metabolic syndrome. Br J Nutr. 2021;125(8):863–875. 32792029 10.1017/S0007114520003177

[49] Liebig M , Dannenberger D, Vollmar B, Abshagen K. Endogenously increased n-3 PUFA levels in fat-1 transgenic mice do not protect from non-alcoholic steatohepatitis. Hepatobiliary Surg Nutr. 2019;8(5):447–458. 31673534 10.21037/hbsn.2019.04.03 PMC6791993

[50] Kim E-H , Bae J-S, Hahm KB, Cha J-Y. Endogenously synthesized n-3 polyunsaturated fatty acids in fat-1 mice ameliorate high-fat diet-induced non-alcoholic fatty liver disease. Biochem Pharmacol. 2012;84(10):1359–1365. 22981383 10.1016/j.bcp.2012.08.029

[51] Guo X-F , Gao J-L, Li J-M, Li D. Fat-1 mice prevent high-fat plus high-sugar diet-induced non-alcoholic fatty liver disease. Food Funct. 2017;8(11):4053–4061. 28972610 10.1039/c7fo01050h

[52] Sears B , Saha AK. Dietary control of inflammation and resolution. Front Nutr. 2021;8:709435. 34447777 10.3389/fnut.2021.709435 PMC8382877

[53] Rudkowska I , Paradis A-M, Thifault E et al. Transcriptomic and metabolomic signatures of an n-3 polyunsaturated fatty acids supplementation in a normolipidemic/normocholesterolemic Caucasian population. J Nutr Biochem. 2013;24(1):54–61. 22748805 10.1016/j.jnutbio.2012.01.016

[54] Orešič M , Hyötyläinen T, Kotronen A et al. Prediction of non-alcoholic fatty-liver disease and liver fat content by serum molecular lipids. Diabetologia. 2013;56(10):2266–2274. 23824212 10.1007/s00125-013-2981-2 PMC3764317

[55] Lehmann R , Franken H, Dammeier S et al. Circulating lysophosphatidylcholines are markers of a metabolically benign nonalcoholic fatty liver. Diabetes Care. 2013;36(8):2331–2338. 23514731 10.2337/dc12-1760 PMC3714475

[56] Masoodi M , Gastaldelli A, Hyötyläinen T et al. Metabolomics and lipidomics in NAFLD: biomarkers and non-invasive diagnostic tests. Nat Rev Gastroenterol Hepatol. 2021;18(12):835–856. 34508238 10.1038/s41575-021-00502-9

[57] Gorden DL , Myers DS, Ivanova PT et al. Biomarkers of NAFLD progression: a lipidomics approach to an epidemic. J Lipid Res. 2015;56(3):722–736. 25598080 10.1194/jlr.P056002 PMC4340319

[58] Vance DE , Vance JE., eds. CHAPTER 8 - Phospholipid biosynthesis in eukaryotes. In: Biochemistry of Lipids, Lipoproteins and Membranes. 5thed. San Diego, CA: Elsevier, 2008:213–244.

[59] Puri P , Wiest MM, Cheung O et al. The plasma lipidomic signature of nonalcoholic steatohepatitis. Hepatology. 2009;50(6):1827–1838. 19937697 10.1002/hep.23229 PMC5031239

[60] Heymans HSA , Bosch HVD, Schutgens RBH, Tegelaers WHH, Walther J-U, Müller-Höcker J, Borst P. Deficiency of plasmalogens in the cerebro-hepato-renal (Zellweger) syndrome. Eur J Pediatr. 1984;142(1):10–15. 6714253 10.1007/BF00442582

[61] Li Z , Agellon LB, Allen TM et al. The ratio of phosphatidylcholine to phosphatidylethanolamine influences membrane integrity and steatohepatitis. Cell Metab. 2006;3(5):321–331. 16679290 10.1016/j.cmet.2006.03.007

[62] Gaposchkin DP , Farber HW, Zoeller RA. On the importance of plasmalogen status in stimulated arachidonic acid release in the macrophage cell line RAW 264.7. Biochim Biophys Acta. 2008;1781(4):213–219. 18328831 10.1016/j.bbalip.2008.01.007

[63] Silvestri C , Diâ Marzo V. The endocannabinoid system in energy homeostasis and the etiopathology of metabolic disorders. Cell Metab. 2013;17(4):475–490. 23562074 10.1016/j.cmet.2013.03.001

[64] Osei-Hyiaman D , Depetrillo M, Pacher P et al. Endocannabinoid activation at hepatic CB1 receptors stimulates fatty acid synthesis and contributes to diet-induced obesity. J Clin Invest. 2005;115(5):1298–1305. 15864349 10.1172/JCI23057 PMC1087161

[65] Liu J , Zhou L, Xiong K et al. Hepatic cannabinoid receptor-1 mediates diet-induced insulin resistance via inhibition of insulin signaling and clearance in mice. Gastroenterology. 2012;142(5):1218–1228.e1. 22307032 10.1053/j.gastro.2012.01.032 PMC3482511

[66] Bidu Cã©L , Escoula Q, Bellenger S et al. The transplantation of ω3 PUFA-altered gut microbiota of fat-1 mice to wild-type littermates prevents obesity and associated metabolic disorders. Diabetes. 2018;67(8):1512–1523. 29793999 10.2337/db17-1488

[67] Kaliannan K , Wang B, Li X-Y, Bhan AK, Kang JX. Omega-3 fatty acids prevent early-life antibiotic exposure-induced gut microbiota dysbiosis and later-life obesity. Int J Obes (Lond). 2016;40(6):1039–1042. 26876435 10.1038/ijo.2016.27

[68] Wang S , Zhu Q, Liang G, Franks T et al. Cannabinoid receptor 1 signaling in hepatocytes and stellate cells does not contribute to NAFLD. J Clin Invest. 2021;131(22):10.1172/JCI152242 PMC8592555 34499619

[69] Cani PD , Geurts L, Matamoros S, Plovier H, Duparc T. Glucose metabolism: focus on gut microbiota, the endocannabinoid system and beyond. Diabetes Metab. 2014;40(4):246–257. 24631413 10.1016/j.diabet.2014.02.004

[70] Balvers MGJ , Verhoeckx KCM, Plastina P, Wortelboer HM, Meijerink J, Witkamp RF. Docosahexaenoic acid and eicosapentaenoic acid are converted by 3T3-L1 adipocytes to N-acyl ethanolamines with anti-inflammatory properties. Biochim Biophys Acta. 2010;1801(10):1107–1114. 20601112 10.1016/j.bbalip.2010.06.006

[71] Park S-W , Hah JH, Oh S-M, Jeong W-J, Sung M-W. 5-lipoxygenase mediates docosahexaenoyl ethanolamide and N-arachidonoyl-L-alanine-induced reactive oxygen species production and inhibition of proliferation of head and neck squamous cell carcinoma cells. BMC Cancer. 2016;16(1):458. 27411387 10.1186/s12885-016-2499-3 PMC4942960

[72] Sugiura T , Kondo S, Kishimoto S et al. Evidence that 2-arachidonoylglycerol but not N-palmitoylethanolamine or anandamide is the physiological ligand for the cannabinoid CB2 receptor. Comparison of the agonistic activities of various cannabinoid receptor ligands in HL-60 cells. J Biol Chem. 2000;275(1):605–612. 10617657 10.1074/jbc.275.1.605

[73] Alharthi N , Christensen P, Hourani W et al. n-3 polyunsaturated N-acylethanolamines are CB(2) cannabinoid receptor-preferring endocannabinoids. Biochim Biophys Acta Mol Cell Biol Lipids. 2018;1863(11):1433–1440. 30591150 10.1016/j.bbalip.2018.08.003

[74] Mallat A , Teixeira-Clerc F, Lotersztajn S. Cannabinoid signaling and liver therapeutics. J Hepatol. 2013;59(4):891–896. 23567085 10.1016/j.jhep.2013.03.032

[75] Turcotte C , Blanchet M-R, Laviolette M, Flamand N. The CB(2) receptor and its role as a regulator of inflammation. Cell Mol Life Sci. 2016;73(23):4449–4470. 27402121 10.1007/s00018-016-2300-4 PMC5075023

[76] Yu H-N , Zhu J, Pan W-S, Shen S-R, Shan W-G, Das UN. Effects of fish oil with a high content of n-3 polyunsaturated fatty acids on mouse gut microbiota. Arch Med Res. 2014;45(3):195–202. 24681186 10.1016/j.arcmed.2014.03.008

[77] Hakimian JK , Dong TS, Barahona JA et al. Dietary supplementation with omega-3 polyunsaturated fatty acids reduces opioid-seeking behaviors and alters the gut microbiome. Nutrients. 2019;11(8):1900. 31416242 10.3390/nu11081900 PMC6723154

[78] Everard A , Lazarevic V, Gaïa N et al. Microbiome of prebiotic-treated mice reveals novel targets involved in host response during obesity. ISME J. 2014;8(10):2116–2130. 24694712 10.1038/ismej.2014.45 PMC4163056

[79] Meng Yu , Li X, Zhang J, Wang C, Lu F. Effects of different diets on microbiota in the small intestine mucus and weight regulation in rats. Sci Rep. 2019;9(1):8500. 31186491 10.1038/s41598-019-44994-7 PMC6560036

[80] Patankar JV , Wong CK, Morampudi V et al. Genetic ablation of Cyp8b1 preserves host metabolic function by repressing steatohepatitis and altering gut microbiota composition. Am J Physiol Endocrinol Metab. 2018;314(5):E418–E432. 29066462 10.1152/ajpendo.00172.2017 PMC6008057

[81] Zhang Xu , Zhao Y, Xu J et al. Modulation of gut microbiota by berberine and metformin during the treatment of high-fat diet-induced obesity in rats. Sci Rep. 2015;5(1):14405. 26396057 10.1038/srep14405 PMC4585776

[82] Martínez I , Wallace G, Zhang C et al. Diet-induced metabolic improvements in a hamster model of hypercholesterolemia are strongly linked to alterations of the gut microbiota. Appl Environ Microbiol. 2009;75:4175–4184. 19411417 10.1128/AEM.00380-09 PMC2698331

[83] Lin C-H , Chen Y-H, Tsai T-Y, Pan T-M. Effects of deep sea water and *Lactobacillus paracasei* subsp. *paracasei* NTU 101 on hypercholesterolemia hamsters gut microbiota. Appl Microbiol Biotechnol. 2017;101(1):321–329. 27709286 10.1007/s00253-016-7868-y

[84] Geurts L , Everard A, Van Hul M et al. Adipose tissue NAPE-PLD controls fat mass development by altering the browning process and gut microbiota. Nat Commun. 2015;6(1):6495. 25757720 10.1038/ncomms7495 PMC4382707

[85] Pontis S , Ribeiro A, Sasso O, Piomelli D. Macrophage-derived lipid agonists of PPAR-α as intrinsic controllers of inflammation. Crit Rev Biochem Mol Biol. 2016;51(1):7–14. 26585314 10.3109/10409238.2015.1092944

[86] Lefort C , Roumain M, Van Hul M et al. Hepatic NAPE-PLD is a key regulator of liver lipid metabolism. Cells. 2020;9(5):1247. 32443626 10.3390/cells9051247 PMC7291298

[87] Chambers ES , Viardot A, Psichas A et al. Effects of targeted delivery of propionate to the human colon on appetite regulation, body weight maintenance and adiposity in overweight adults. Gut. 2015;64(11):1744–1754. 25500202 10.1136/gutjnl-2014-307913 PMC4680171

[88] Pujo J , Petitfils C, Le Faouder P et al. Bacteria-derived long chain fatty acid exhibits anti-inflammatory properties in colitis. Gut. 2021;70(6):1088–1097. 32978245 10.1136/gutjnl-2020-321173

[89] Liu Y , Luo Y, Wang X, Luo L, Sun K, Zeng L. Gut microbiome and metabolome response of pu-erh tea on metabolism disorder induced by chronic alcohol consumption. J Agric Food Chem. 2020;68(24):6615–6627. 32419453 10.1021/acs.jafc.0c01947

[90] Chayanupatkul M , Somanawat K, Chuaypen N et al. Probiotics and their beneficial effects on alcohol-induced liver injury in a rat model: the role of fecal microbiota. BMC Complement Med Ther. 2022;22(1):168. 35733194 10.1186/s12906-022-03643-9 PMC9215017

[91] Thacker PA , Salomons MO, Aherne FX, Milligan LP, Bowland JP. Influence of propionic acid on the cholesterol metabolism of pigs fed hypercholesterol diets. Can J Anim Sci. 1981;61(4):969–975.

[92] Chen W-JL , Anderson JW, Jennings D. Propionate may mediate the hypocholesterolemic effects of certain soluble plant fibers in cholesterol-fed rats. Proc Soc Exp Biol Med. 1984;175(2):215–218. 6320209 10.3181/00379727-175-41791

[93] Seth EC , Taga ME. Nutrient cross-feeding in the microbial world. Front Microbiol. 2014;5:350. 25071756 10.3389/fmicb.2014.00350 PMC4086397

[94] Belenguer A , Duncan SH, Calder AG et al. Two routes of metabolic cross-feeding between *Bifidobacterium adolescentis* and butyrate-producing anaerobes from the human gut. Appl Environ Microbiol. 2006;72(5):3593–3599. 16672507 10.1128/AEM.72.5.3593-3599.2006 PMC1472403

[95] Jakobsson HE , Rodríguez-Piñeiro AM, Schütte A et al. The composition of the gut microbiota shapes the colon mucus barrier. EMBO Rep. 2015;16(2):164–177. 25525071 10.15252/embr.201439263 PMC4328744

