# Supplementary material for: Comparing Transgenic Production to Supplementation of ω-3 PUFA Reveals Distinct But Overlapping Mechanisms Underlying Protection Against Metabolic and Hepatic Disorders
Source: Function (Oxf). 2022 Dec 29;4(2):zqac069. doi: 10.1093/function/zqac069 (PMC9909367; doi:10.1093/function/zqac069)
Supplement: zqac069_Supplemental_File [file zqac069_supplemental_file.docx]

**Dietary model**

# A

**Insuline tolerance test**

**15 D: p=.06 T: p<.001 DxT: p=.04**


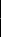

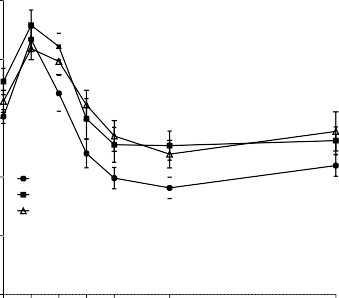

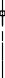


LF ω6 HF ω6

HF ω3

$

**B**

**AUC ipITT**


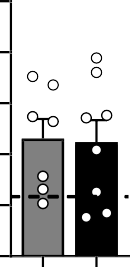
**800**

**12**

**700**

**Glycemia (mmol/L)**

**9**

**600**

**AU**

**6**

**500**

**400**

**3**

**0**

**0 5 10 15 20**

**30 60**

**Time (min)**

**300**

**HF HF**

ω**6** ω**3**

# C D E

**Glucose tolerance test**

**20 D: p=.09 T: p<.001 DxT: p<.001**


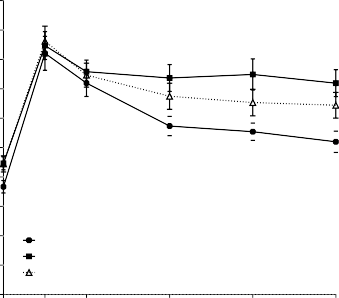

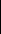

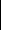

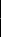


$

$$

$

LF ω6

HF ω6 HF ω3

**18**

**16**

**14**

**Glycemia (mmol/L)**

**12**

**10**

**8**

**6**

**4**

**2**

**0**

**Fasting glycemia oGTT**


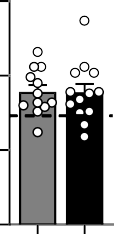
**15**

**Glycemia (mmol/L)**

**10**

$

**5**

**0**

**2500**

**2000**

**1500**

**AU**

**1000**

**500**

**0**

**AUC oGTT**


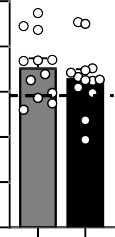


**0 15**

**30 60 90 120**

**Time (min)**

**HF HF**

ω**6** ω**3**

**HF HF**

ω**6** ω**3**

# F

**3.5**


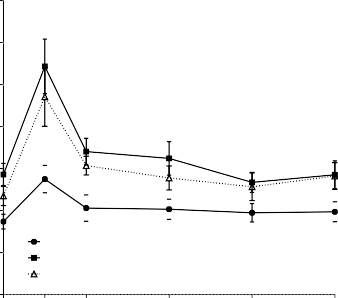

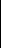

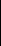

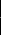

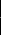

$$$

$$

$$

$*p=.06*

$

$$

LF ω6 HF ω6

HF ω3

**Glucose-stimulated insulin secretion**

**D: p=.002 T: p<.001** DxT: p=.26

**G**

**Fasting insulinemia oGTT**

**H I**

**AUC GSIS**

**HOMA-IR**

**3.0**

**2.5**

**Glycemia (mmol/L)**

**2.0**

**1.5**

**3 400 40**


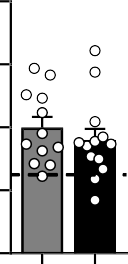

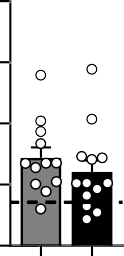

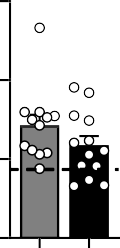
**300 30**

**Glycemia (mmol/L)**

**HOMA-IR Index**

**2**

**200 20**

**AU**

**1.0**

**0.5**

**0.0**

**0**

**15 30 60 90 120**

**Time (min)**

**1**

**0**

**HF HF**

ω**6** ω**3**

**100**

**0**

**HF HF**

ω**6** ω**3**

**10**

**0**

**HF HF**

ω**6** ω**3**

**Supplemental Fig S1**. **The ω-3 PUFA supplementation does not improves glucose homeostasis**. (**A**) Glycemic response and (**B**) corresponding area under the curve (AUC) to the insulin tolerance test (ipITT) performed at week 10. (**C**) Glycemic response (**D**) fasting glycaemia and (**E**) AUC before and during the glucose tolerance test (oGTT) performed at week 12. (**F**) Glucose-stimulated insulin secretion

(**G**) fasting insulin and (**H**) AUC before and during the oGTT. (**I**) Homeostasis model assessment of insulin resistance (HOMA-IR). Values are means ± SEM of n=9-13 mice per group. One-way ANOVA followed by Dunnett *post hoc* test compared to HF-ω6 group. Dotted line represents LF-ω6 group as a reference. *P<.05, **P<.01, ***P<.001.

#
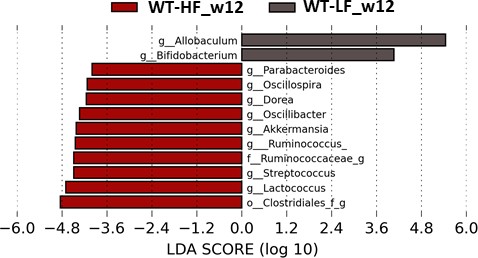

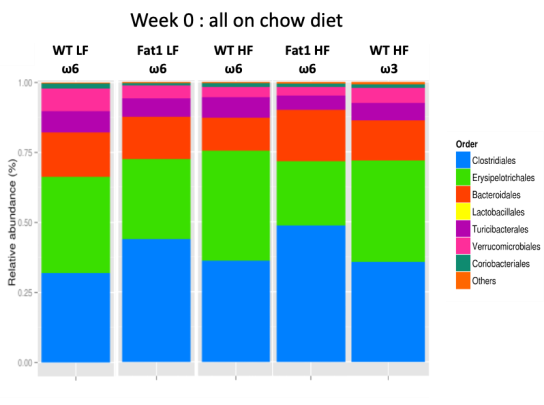
A B

## Genetic model C

**Fecal Lcn2 D**

**Caecum**

**Total cecal SCFA**

**E**

D: p=.58 G: p=.11 DxG: p=.15


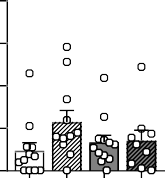
**20**

**15**

**ng/g feces**

**10**

**5**

**D: p=.005** G: p=.86 DxG: p=.87

**0.6**


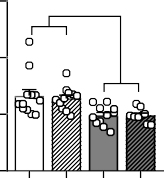


✱

**0.4**

**Weight (g)**

**0.2**

D: p=.13 G: p=.98 DxG: p=.94


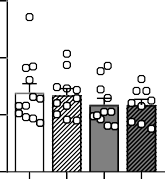
**1500**

**µg/g caecal content**

**1000**

**500**

**0**

**WT Fat-1 WT Fat-1**

**0.0**

**WT Fat-1 WT Fat-1**

**0**

**WT Fat-1 WT Fat-1**

**LF LF HF HF**

**LF LF HF HF**

**LF LF HF HF**

# F

**Acetate**

**G H**

**Propionate**

**I**

**Butyrate**

**J**

**Valerate**

**Isovalerate**

**K**

**Isobutyrate**

D: p=.63 G: p=.77 DxG: p=.70

**D: p=.003** G: p=.14 DxG: p=.35

D: p=.90 G: p=.75 DxG: p=.97

**D: p=.02** G: p=.60 DxG: p=.54

**D: p<.001** G: p=.76 DxG: p=.74

**D: p<.001** G: p=.96 DxG: p=.95

**800**

**µg/g caecal content**

**600**

**400**

**200**

**0**


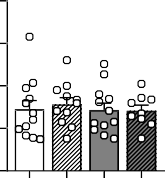


**WT Fat-1 WT Fat-1**

**350**

**300**

**µg/g caecal content**

**250**

**200**

**150**

**100**

**50**

**0**

✱✱


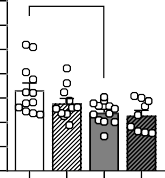


**WT Fat-1 WT Fat-1**

**400**

**300**

**µg/g caecal content**

**200**

**100**

**0**


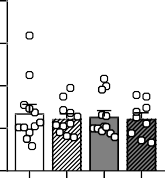


**WT Fat-1 WT Fat-1**

**70**

**60**


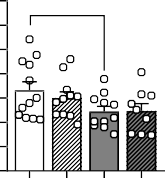


✱

**µg/g caecal content**

**50**

**40**

**30**

**20**

**10**

**0**

**WT Fat-1 WT Fat-1**

✱✱✱

**80**


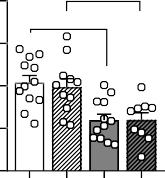


✱✱

**µg/g caecal content**

**60**

**40**

**20**

**0**

**WT Fat-1 WT Fat-1**

**60**

**50**


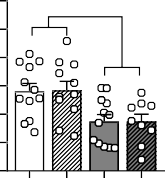


✱✱

**µg/g caecal content**

**40**

**30**

**20**

**10**

**0**

**WT Fat-1 WT Fat-1**

**LF LF**

**HF HF**

**LF LF**

**HF HF**

**LF LF**

**HF HF**

**LF LF**

**HF HF**

**LF LF**

**HF HF**

**LF LF**

**HF HF**

## Dietary model

1. **Fecal Lcn2**
2. **Caecum**

**N Total cecal SCFA**


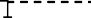

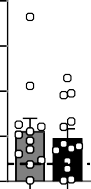
**12 2**

**1**


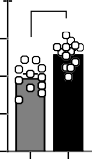
**0.4** ✱

**9**

**ng/g feces**

**Weight (g)**

**0.3**

**6**

**0.2**

**3 0.1**

**1500**


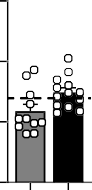
**1000**

**µg/g caecal content**

**500**

**0**

**HF HF**

**O P** ω**6** ω**3 Q**

**0.0**

**HF HF**

ω**6** ω**3 R**

**0**

**HF HF**

ω**6** ω**3 S T**

**600**

**µg/g caecal content**

**400**

**200**

**Acetate**

**300**

**µg/g caecal content**

**200**

**100**

**Propionate**


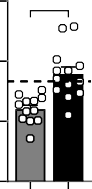
✱✱

**250**

**µg/g caecal content**

**200**

**150**

**100**

**50**

**Butyrate**

**Valerate**


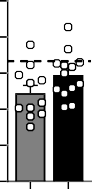
**50**

**µg/g caecal content**

**40**

**30**

**20**

**10**

**150**

**100**

**µg/g caecal content**

**50**

**Isovalerate**


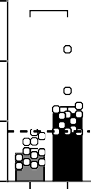
✱✱✱

**Isobutyrate**


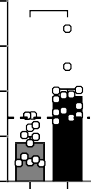
**80** ✱✱✱

**µg/g caecal content**

**60**

**40**

**20**


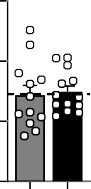
**0**

**HF HF**

ω**6** ω**3**

**0**

**HF HF**

ω**6** ω**3**

**0**


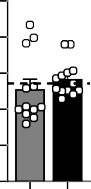
**HF HF**

ω**6** ω**3**

**0**

**HF HF**

ω**6** ω**3**

**0**

**HF HF**

ω**6** ω**3**

**0**

**HF HF**

ω**6** ω**3**

**Supplemental Fig. S2**. **Impact of endogenous production and ω-3 PUFA supplementation on intestinal health**. (**A**) Stacked bar plot representing the bacterial relative abundance before treatment (all animals on chow diet) at order level, (**B**) LEfSe analysis identifying significant differentially genera abundance between WT-HF and WT-LF mice after 12 weeks. Panels (**C-K**) refer to the genetic model and (**L**-**T**) to the dietary model. (**C**, **L**) Fecal lipocalin-2. (**D**, **M**) and (**E**, **N**) total caecal short chain fatty acid (SCFA) content. (**F**, **O**) acetate (**G**, **P**) propionate (**H**, **Q**) butyrate (**I**, **R**) valerate (**J**, **S**) isovalerate and (**K**, **T**) isobutyrate caecal concentrations. Values are means ± SEM of n=9-13 mice per group. For the genetic model: two-way ANOVA followed by Tukey *post hoc* test. P-values of main effects for diet (D) and gene (G) or diet x gene (DxG) interaction are recorded under the title of each graph. For the dietary model: one-way ANOVA followed by Dunnett *post hoc* test compared to HF-ω6 group. Dotted line represents LF-ω6 group as a reference. *P<.05, **P<.01, ***P<.001. “f” and “g” at the end of taxon denote unclassified family and genus, respectively.

**Genetic model**

# A B C

**ALT**

**AST**

**Plasma NEFA**

**D: p=.06** G: p=.37 DxG: p=.73

**D: p=.001** G: p=.34 DxG: p=.37

D: p=.45 G: p=.94 DxG: p=.89


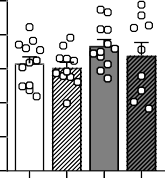
**1.0**

**0.8**

**0.6**

**mEq/L**

**0.4**

**0.2**

**0.0**

**200**

**150**

**100**

**U/L**

**50**

**0**

*p=0.05*

**WT Fat-1 WT Fat-1**


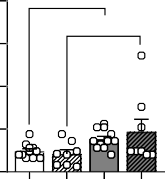


✱✱

**160**

**140**

**120**

**100**

**U/L**

**80**

**60**

**40**

**20**

**0**


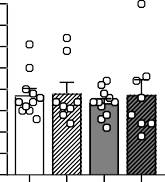


**WT Fat-1 WT Fat-1**

**WT Fat-1 WT Fat-1**

**LF LF**

**HF HF**

**LF LF**

**HF HF**

**LF LF HF HF**

## Dietary model

**D Plasma NEFA**

**E ALT**

**F AST**

**1.0**

*p=.08*

*p=.06*

**60**

**100**

*p=.07*

**0.8 80**

**40**

**mEq/L**

**0.6 60**

**U/L**

**U/L**

**0.4 40**

**20**

**0.2 20**

**0.0**

**HF HF**

ω**6** ω**3**

**0**

**HF HF**

ω**6** ω**3**

**0**

**HF HF**

ω**6** ω**3**

**Supplemental Fig. S3**. **ω3-PUFA supplementation exerts a greater effect improving lipids and reducing hepatic markers of acute injury**. Panels (**A-C**) refer to the genetic model and (**D-F**) to the dietary model. Plasma (**A**, **D** non- esterified fatty acids (**B**, **E**) alanine aminotransferase (ALT) (**C**, **F**) aspartate aminotransferase (AST). Values are means

± SEM of n=9-13 mice per group. For the genetic model: two-way ANOVA followed by Tukey *post hoc* test. P-values of main effects for diet (D) and gene (G) or diet x gene (DxG) interaction are recorded under the title of each graph. For the dietary model: one-way ANOVA followed by Dunnett *post hoc* test compared to HF-ω6 group. Dotted line represents LF-ω6 group as a reference. Thick dotted line on y axis represents the half of the quantification limit (0.08 pg/mg). *P<.05, **P<.01, ***P<.001.

**Supplemental Table S1**. **Hepatic lipid profile of the five treated groups (µM).** The two last columns show statistical differences referring to the dietary and the genetic models respectively. Dietary model: One-way ANOVA with Tukey *post hoc* test (*vs* HF ω6 group). Significant effects are notified by “diet” (LF WT ω6 *vs* HF ω6) or “gavage” (HF ω3 group *vs* HF ω6). Genetic model: 2-way ANOVA followed by a Tukey *post hoc* test. Significant main effects are notified by “D” (diet) or “G” (gene).

**Hepatic Lipid profile (µM)**

| **LF WT LF Fat-1 HF WT HF Fat-1 HF WT**  **ω6 ω6 ω6 ω6 ω3** | | | | | | | | | | | | | | | | **Dietary model Genetic model**  **One-way ANOVA listing 2-way ANOVA listing** |
| --- | --- | --- | --- | --- | --- | --- | --- | --- | --- | --- | --- | --- | --- | --- | --- | --- |
| **C10:0** | 42.19 | ± | 10.08 | 42.46 | ± | 10.07 | 29.40 | ± | 8.95 | 24.25 | ± | 9.67 | 38.01 | ± | 7.32 | - -  gavage -  diet: p=.09, gavage D, G: p=.07 gavage -  gavage -  diet D, DxG: p=.08  - DxG: p=.08  gavage D, G: p=.07  diet D  diet D  gavage -  gavage D, G  diet, gavage D  diet: p=.06, gavage D, G: p=.05  - -  diet, gavage D, G  gavage -  gavage D, G  diet, gavage D, G:p=.05 diet D  diet D  - -  diet D  diet, gavage D; G:p=.06  - -  gavage G: p=.09  gavage G  gavage G   - D   diet D  diet: p=.07, gavage D, G   - D   diet, gavage D  gavage D: p=.08  gavage D, G, DxG  gavage D: p=.06, G: p=.06  diet, gavage D, G: p=.08  gavage D, G  diet, gavage D  diet, gavage D, G: p=.05  diet, gavage D, G |
| **C14:0** | 37.24 | ± | 3.71 | 30.29 | ± | 4.33 | 39.39 | ± | 5.05 | 30.29 | ± | 6.75 | 16.39 | ± | 1.58 |  |
| **C16:0** | 1269.10 | ± | 101.91 | 1086.40 | ± | 91.54 | 1716.11 | ± | 159.22 | 1381.42 | ± | 195.28 | 1070.63 | ± | 63.97 |  |
| **C18:0** | 332.73 | ± | 15.76 | 313.23 | ± | 13.13 | 306.28 | ± | 5.77 | 321.60 | ± | 29.87 | 358.78 | ± | 15.49 |  |
| **C20:0** | 5.55 | ± | 1.00 | 5.36 | ± | 1.00 | 7.45 | ± | 0.73 | 12.75 | ± | 7.64 | 3.44 | ± | 1.43 |  |
| **C22:0** | 0.34 | ± | 0.25 | 0.08 | ± | 0.08 | 1.90 | ± | 0.46 | 3.03 | ± | 0.61 | 3.57 | ± | 1.58 |  |
| **C24:0** | 9.10 | ± | 1.20 | 5.37 | ± | 1.58 | 8.89 | ± | 1.18 | 9.91 | ± | 1.27 | 6.25 | ± | 1.39 |  |
| **Saturated** | 1736.77 | ± | 123.96 | 1483.18 | ± | 106.18 | 2109.41 | ± | 167.31 | 1783.25 | ± | 219.05 | 1497.13 | ± | 76.70 |  |
| **C14:1n5** | 2.18 | ± | 0.36 | 2.06 | ± | 0.41 | 0.69 | ± | 0.29 | 1.08 | ± | 0.28 | 0.65 | ± | 0.22 |  |
| **C16:1n7** | 187.15 | ± | 20.37 | 152.66 | ± | 23.95 | 55.55 | ± | 18.75 | 62.78 | ± | 25.11 | 34.73 | ± | 6.75 |  |
| **C18:1n7** | 121.13 | ± | 38.84 | 103.90 | ± | 28.19 | 183.88 | ± | 27.07 | 131.00 | ± | 25.30 | 56.46 | ± | 5.08 |  |
| **C18:1n9** | 1136.47 | ± | 126.96 | 806.43 | ± | 132.60 | 1842.84 | ± | 218.68 | 1319.89 | ± | 241.22 | 753.66 | ± | 74.76 |  |
| **C18:1n12** | 196.00 | ± | 60.22 | 261.49 | ± | 58.02 | 2239.89 | ± | 150.66 | 1829.67 | ± | 260.43 | 1216.40 | ± | 75.67 |  |
| **C20:1n9** | 15.11 | ± | 2.84 | 9.62 | ± | 1.84 | 24.66 | ± | 4.96 | 16.22 | ± | 2.93 | 10.90 | ± | 0.86 |  |
| **C20:1n12** | 67.35 | ± | 20.29 | 49.85 | ± | 20.19 | 91.23 | ± | 26.29 | 51.77 | ± | 18.19 | 32.56 | ± | 6.82 |  |
| **C20:1n15** | 43.56 | ± | 2.14 | 36.02 | ± | 6.26 | 154.01 | ± | 11.25 | 119.20 | ± | 12.63 | 84.39 | ± | 5.11 |  |
| **C22:1n9** | 0.81 | ± | 0.81 |  | ND |  |  | ND |  |  | ND |  | 2.56 | ± | 0.95 |  |
| **C24:1n9** | 9.51 | ± | 0.59 | 7.34 | ± | 0.89 | 11.41 | ± | 0.85 | 8.84 | ± | 0.99 | 3.52 | ± | 0.83 |  |
| **Monounsaturated** | 1779.26 | ± | 206.93 | 1429.37 | ± | 197.82 | 4604.17 | ± | 408.95 | 3540.46 | ± | 549.84 | 2195.82 | ± | 155.43 |  |
| **C16:1Tn7** | 151.83 | ± | 16.69 | 151.38 | ± | 14.37 | 884.95 | ± | 175.09 | 650.65 | ± | 136.48 | 480.91 | ± | 75.46 |  |
| **C18:1n7T** | 209.16 | ± | 9.61 | 203.17 | ± | 15.12 | 503.15 | ± | 85.40 | 493.02 | ± | 89.70 | 361.52 | ± | 46.19 |  |
| **C18:1n9T** | 175.10 | ± | 54.11 | 159.56 | ± | 52.27 | 220.55 | ± | 79.48 | 112.83 | ± | 78.70 | 254.99 | ± | 53.55 |  |
| **Trans** | 536.09 | ± | 58.05 | 514.10 | ± | 47.19 | 1608.65 | ± | 185.05 | 1256.51 | ± | 145.61 | 1097.43 | ± | 96.72 |  |
| **C18:2n6** | 509.65 | ± | 39.11 | 455.15 | ± | 28.73 | 816.06 | ± | 56.67 | 659.92 | ± | 87.63 | 361.48 | ± | 27.76 |  |
| **C18:3n6** | 0.77 | ± | 0.77 | 0.56 | ± | 0.56 | 2.47 | ± | 1.35 | 1.23 | ± | 1.23 | 0.50 | ± | 0.34 |  |
| **C20:2n6** | 20.18 | ± | 1.77 | 18.90 | ± | 2.08 | 21.96 | ± | 2.74 | 15.11 | ± | 2.55 | 10.66 | ± | 5.69 |  |
| **C20:3n6** | 49.99 | ± | 1.94 | 44.01 | ± | 1.84 | 53.26 | ± | 1.83 | 43.38 | ± | 3.79 | 32.04 | ± | 1.24 |  |
| **C20:4n6** | 248.45 | ± | 18.33 | 211.56 | ± | 13.29 | 262.08 | ± | 8.52 | 208.18 | ± | 23.97 | 94.20 | ± | 6.19 |  |
| **C22:2n6** | 0.73 | ± | 0.73 | 0.00 | ± | 0.00 | 2.45 | ± | 1.04 | 2.78 | ± | 1.16 | 0.18 | ± | 0.18 |  |
| **C22:4n6** | 8.02 | ± | 0.40 | 7.77 | ± | 0.24 | 3.79 | ± | 0.40 | 4.32 | ± | 0.38 | 4.77 | ± | 0.21 |  |
| **Total ω6** | 837.78 | ± | 56.84 | 737.96 | ± | 43.22 | 1162.08 | ± | 64.37 | 934.93 | ± | 111.33 | 503.83 | ± | 31.97 |  |
| **C18:3n3** | 2.34 | ± | 0.57 | 1.05 | ± | 0.54 | 6.27 | ± | 2.06 | 6.43 | ± | 4.20 | 4.16 | ± | 1.43 |  |
| **C20:3n3** |  | ND |  |  | ND |  | 4.65 | ± | 0.71 | 5.86 | ± | 0.59 | 7.22 | ± | 0.38 |  |
| **C20:5n3** |  | ND |  |  | ND |  | 3.50 | ± | 1.03 | 13.19 | ± | 11.50 | 82.44 | ± | 7.68 |  |
| **C22:5n3** |  | ND |  |  | ND |  | 2.66 | ± | 1.12 | 11.10 | ± | 4.07 | 39.59 | ± | 4.46 |  |
| **C22:6n3** | 32.67 | ± | 2.48 | 39.46 | ± | 2.79 | 39.29 | ± | 2.76 | 65.39 | ± | 20.21 | 178.94 | ± | 19.43 |  |
| **Total ω3** | 35.01 | ± | 2.94 | 40.51 | ± | 3.16 | 56.36 | ± | 2.96 | 101.96 | ± | 35.68 | 312.35 | ± | 31.45 |  |
| **ω6/ω3** | 24.61 | ± | 1.23 | 18.56 | ± | 0.60 | 20.77 | ± | 0.95 | 13.91 | ± | 2.08 | 1.71 | ± | 0.10 |  |
| **C20:3n9** | 7.94 | ± | 3.42 | 7.67 | ± | 3.14 | 24.71 | ± | 2.70 | 20.34 | ± | 3.47 | 0.68 | ± | 0.68 |  |
| **PUFA** | 880.74 | ± | 61.74 | 786.15 | ± | 47.85 | 1243.15 | ± | 66.60 | 1057.24 | ± | 103.67 | 816.86 | ± | 60.46 |  |
| **Total Fatty acids** | 4932.86 | ± | 385.87 | 4212.80 | ± | 315.76 | 9565.38 | ± | 697.54 | 7637.46 | ± | 919.33 | 5607.24 | ± | 287.42 |  |

**Genetic model**

# A B C

**Ether linked PC**

D: p=.0001 G: p=.02 DxG: p=.05

✱

**6**


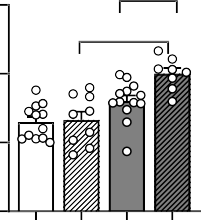


✱✱✱

**Ether linked PE**

D: p=.0003 G: p=.005 DxG: p=.70

✱

**4**


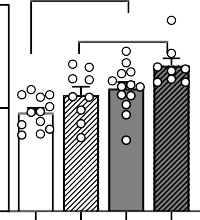


✱

**PC:PE**

D: p=.0003 G: p=.20 DxG: p=.29

**1.5**


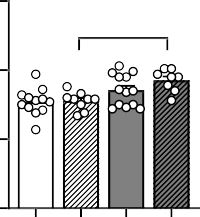


✱

**4 1.0**

**pmol/g liver**

**pmol/g liver**

**Ratio**

**2**

**2 0.5**

**0**

**WT Fat-1 WT**

**Fat-1**

**0**

**WT Fat-1 WT**

**Fat-1**

**0.0**

**WT Fat-1 WT**

**Fat-1**

**LF LF**

**HF HF**

**LF LF**

**HF HF**

**LF LF**

**HF HF**

**D 17-HDHA E**

**18-HEPE**

**D: p=.05** G: p=.28 **DxG: p=.07**

**6**


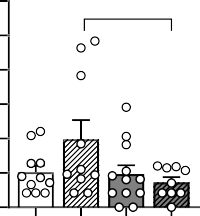


p=.07

**5**

**4**

**pg/mg**

**3**

**2**

**1**

**0**

D: p=.11 G: p=.32 DxG: p=.32


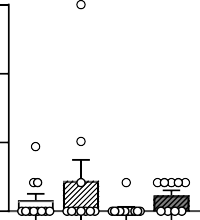
**0.6**

**0.4**

**pg/mg**

**0.2**

**0.0**

**WT Fat-1 WT Fat-1**

**WT Fat-1 WT Fat-1**

**LF LF HF HF**

**LF LF HF HF**

## Dietary model

**F G Ether linked PE H**

**Ether linked PC**


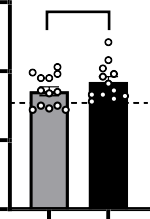
**PC:PE**

✱✱✱

**8 5**


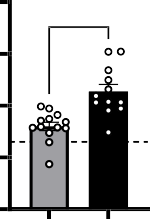


✱✱✱✱

**4**

**6**

**pmol/g liver**

**pmol/g liver**

**3**

**4**

**2**

**2 1**

**1.5**

**1.0**

**Ratio**

**0.5**

✱✱✱✱

**0**

**HF HF**

ω**6** ω**3**

**0**

**HF HF**

ω**6** ω**3**

**0.0**

**HF HF**

ω**6** ω**3**

**I 17-HDHA**

✱✱✱

**12**


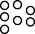


**9**

**pg/mg**

**6**

**3**

**J 18-HEPE**

✱✱✱


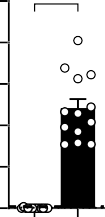
**10**

**8**

**6**

**pg/mg**

**4**

**2**

**K**

**0.25**

**0.20**

**PDX**

**(pg/mg tissue)**

**0.15**

**0.10**

**0.05**

**PDX**


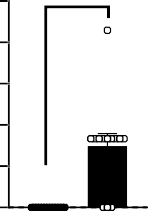
✱✱✱✱

**0**

**HF HF**

ω**6** ω**3**

**0**

**HF HF**

ω**6** ω**3**

**0.00**

**WTHF HFw3**

**Supplemental Fig. S4**. **ω3-PUFA supplementation exert a greater effect improving lipemia and reducing hepatic steatosis compared to *fat-1* endogenous production**. Panels (**A-E**) refer to the genetic model and (**F-K**) to the dietary model. Hepatic (**A**, **G**) total ether linked PC (**B**, **H**) total ether linked PE (**C**, **H**) Total PC to total PE ratio. **(D,I)** DHA derived 17-HDHA, **(E,J)** EPA derived 18-HEPE and **(K)** 17-HDHA derived protectin-DX (PDX). Values are means ± SEM of n=9-13 mice per group. For the genetic model: two-way ANOVA followed by Tukey *post hoc* test. P-values of main effects for diet (D) and gene (G) or diet x gene (DxG) interaction are recorded under the title of each graph. For the dietary model: one-way ANOVA followed by Dunnett *post hoc* test compared to HF-ω6 group. Dotted line represents LF-ω6 group as a reference. *P<.05, **P<.01, ***P<.001.

**Supplemental Table S2**. **Hepatic PC profile (pmol/g liver).** The two last columns show statistical differences referring to the dietary and the genetic models respectively. Dietary model: One-way ANOVA with Tukey *post hoc* test (*vs* HF ω6 group). Significant effects are notified by “diet” (LF WT ω6 *vs* HF ω6) or “gavage” (HF ω3 group *vs* HF ω6). Genetic model: 2-way ANOVA followed by a Tukey *post hoc* test. Significant main effects are notified by “D” (diet) or “G” (gene).

|  | LF WT  ω6 | LF Fat 1 ω6 | HF WT  ω6 | HF Fat 1 ω6 | HF WT  ω3 | Dietary Model One-way ANOVA | Genetic Model Two-way ANOVA |
| --- | --- | --- | --- | --- | --- | --- | --- |
| PC 30:1 | 0.38 ± 0.31 | 0.45 ± 0.11 | 0.49 ± 0.04 | 1.25 ± 0.06 | 3.00 ± 0.17 | Gavage | D,G,DXG |
| PC 30:0 | 0.22 ± 0.01 | 0.27 ± 0.02 | 0.16 ± 0.01 | 0.22 ± 0.01 | 0.43 ± 0.03 | Gavage | D,G |
| PC 32:2 | 0.63± 0.04 | 0.77 ± 0.10 | 0.73 ± 0.04 | 0.99± 0.07 | 1.77 ± 0.11 | Gavage | D,G |
| PC 32:1 | 2.83 ± 0.20 | 3.46 ± 0.33 | 2.63 ± 0.14 | 3.60 ± 0.20 | 7.77 ± 0.43 | Gavage | G |
| PC 32:0 | 2.94 ± 0.17 | 3.56 ± 0.33 | 3.33 ± 0.20 | 4.79 ± 0.25 | 8.50 ± 0.15 | Gavage | D,G |
| PC 34:4 | 0.50 ± 0.03 | 0.55 ± 0.06 | 0.86 ± 0.06 | 1.10 ± 0.06 | 0.97 ± 0.06 | Diet | D,G |
| PC 34:3 | 4.36 ± 0.26 | 5.54 ± 0.56 | 9.08 ± 0.55 | 12.3 ± 0.67 | 16.2 ± 0.89 | Diet, Gavage | D,G |
| PC 34:2 | 34.7 ± 1.83 | 45.6 ± 3.88 | 55.8 ± 3.51 | 76.1 ± 3.80 | 114 ± 6.18 | Diet, Gavage | D,G |
| PC 34:1 | 28.3 ± 1.75 | 29.8 ± 2.27 | 32.7 ± 1.57 | 43.0 ± 2.02 | 83.9 ± 4.83 | Gavage | D.G.DXG |
| PC 34:0 | 2.72 ± 0.15 | 2.87 ± 0.24 | 3.11 ± 0.16 | 4.04 ± 0.17 | 7.96 ± 0.43 | Gavage | D.G.DXG |
| PC 35:2 | 0.38 ± 0.02 | 0.43 ± 0.04 | 0.59 ± 0.04 | 0.78 ± 0.06 | 1.07 ± 0.06 | Diet, Gavage | D,G |
| PC 38:5 | 21.4 ± 1.57 | 20.5 ± 1.80 | 40.2 ± 2.62 | 43.2 ± 2.50 | 43.3 ± 2.77 | Diet | D |
| PC 38:4 | 28.5 ± 1.90 | 28.5 ± 2.84 | 41.9 ± 2.49 | 48.0 ± 2.19 | 23.4 ± 1.72 | Diet, Gavage | D |
| PC 38:3 | 7.18 ± 0.49 | 6.72 ± 0.78 | 9.98 ± 0.53 | 11.2 ± 0.42 | 7.76 ± 0.47 | Diet, Gavage | D |
| PC 40:7 | 2.59 ± 0.18 | 3.36 ± 0.35 | 5.06 ± 0.32 | 7.40 ± 0.33 | 28.3 ± 2.07 | Gavage | D,G,DXG |
| PC 40:6 | 3.89 ± 0.27 | 4.64 ± 0.55 | 6.41 ± 0.42 | 8.38 ± 0.40 | 31.5 ± 2.26 | Gavage | D,G |
| PC 40:5 | 2.95 ± 0.23 | 2.68 ± 0.35 | 4.19 ± 0.31 | 4.31 ± 0.26 | 6.16 ± 0.38 | Diet, Gavage | D |
| PC 40:4 | 0.87 ± 0.06 | 0.81 ± 0.09 | 1.21 ± 0.07 | 1.30 ± 0.07 | 1.02 ± 0.05 | Diet | D |
| PC 40:3 | 0.23 ± 0.02 | 0.26 ± 0.03 | 0.26 ± 0.01 | 0.37 ± 0.02 | 0.20 ± 0.01 | Gavage | D,G,DXG |
| PC O34:1 | 0.32 ± 0.02 | 0.36 ± 0.04 | 0.43 ± 0.02 | 0.57 ± 0.02 | 0.58 ± 0.04 | Diet, Gavage | D,G |
| PC O36:4 | 0.24 ± 0.02 | 0.25 ± 0.02 | 0.27 ± 0.02 | 0.33 ± 0.02 | 0.16 ± 0.01 | Gavage | D |
| PC O36:3 | 0.17 ± 0.01 | 0.20 ± 0.03 | 0.26 ± 0.02 | 0.32 ± 0.02 | 0.41 ± 0.02 | Diet, Gavage | D,G |
| PC O36:2 | 0.48 ± 0.02 | 0.48 ± 0.04 | 0.61 ± 0.03 | 0.76 ± 0.03 | 1.08 ± 0.06 | Diet, Gavage | D,G,DXG |
| PC O36:1 | 0.25 ± 0.02 | 0.23 ± 0.02 | 0.25 ± 0.01 | 0.33 ± 0.01 | 0.54 ± 0.04 | Gavage | D,DXG |
| PC O38:4 | 0.49 ± 0.03 | 0.51 ± 0.05 | 0.58 ± 0.03 | 0.70 ± 0.02 | 0.47 ± 0.04 | ----- | D |
| PC O38:3 | 0.21 ± 0.02 | 0.17 ± 0.02 | 0.20 ± 0.01 | 0.23 ± 0.01 | 0.21 ± 0.02 | ----- | DXG |

**Supplemental Table S3**. **Hepatic PE profile (%).** The two last columns show statistical differences referring to the dietary and the genetic models respectively. Dietary model: One-way ANOVA with Tukey *post hoc* test (*vs* HF ω6 group). Significant effects are notified by “diet” (LF WT ω6 *vs* HF ω6) or “gavage” (HF ω3 group *vs* HF ω6). Genetic model: 2-way ANOVA followed by a Tukey *post hoc* test. Significant main effects are notified by “D” (diet) or “G” (gene).

|  |  |  |  |  |  | Dietary Model | Genetic |
| --- | --- | --- | --- | --- | --- | --- | --- |
|  | LF WT | LF Fat 1 | HF WT | HF Fat 1 | HF WT | One-way | Model |
|  | ω6 | ω6 | ω6 | ω6 | ω3 | ANOVA | Two-way |
|  |  |  |  |  |  |  | ANOVA |
| PE 34:2 | 8.71 ± 0.60 | 10.3 ± 1.03 | 13.1 ± 0.75 | 15.6 ± 0.92 | 16.2 ± 1.28 | Diet, Gavage | D,G |
| PE 34:1 | 2.20 ± 0.12 | 2.33 ± 0.20 | 2.61 ± 0.13 | 3.11 ± 0.20 | 3.85 ± 0.24 | Gavage | D,G |
| PE 34:0 | 0.26 ± 0.02 | 0.25 ± 0.03 | 0.30 ± 0.02 | 0.35 ± 0.02 | 0.48 ± 0.03 | Gavage | D |
| PE 36:5 | 2.09 ± 0.11 | 2.33 ± 0.20 | 2.42 ± 0.15 | 2.65 ± 0.18 | 15.0 ± 0.71 | Gavage | D |
| PE 36:4 | 21.1 ± 0.81 | 22.0 ± 1.19 | 21.2 ± 1.14 | 23.1 ± 1.38 | 12.0 ± 0.89 | Gavage | ---- |
| PE 36:3 | 9.50 ± 0.54 | 9.84 ± 0.81 | 15.9 ± 0.89 | 17.6 ± 0.86 | 15.1 ± 1.20 | Diet | D |
| PE 36:2 | 7.00 ± 0.25 | 7.53 ± 0.55 | 11.5 ± 0.61 | 13.5 ± 0.72 | 16.5 ± 1.22 | Diet, Gavage | D, G |
| PE 36:1 | 1.36 ± 0.06 | 1.35 ± 0.09 | 1.84 ± 0.10 | 2.18 ± 0.11 | 3.23 ± 0.21 | Diet, Gavage | D |
| PE 38:7 | 0.95 ± 0.04 | 1.45 ± 0.10 | 2.13 ± 0.15 | 3.19 ± 0.18 | 10.0 ± 0.59 | Diet, Gavage | D,G, DXG |
| PE 38:6 | 17.3 ± 0.81 | 24.1 ± 1.53 | 22.4 ± 1.42 | 31.4 ± 1.85 | 105 ± 5.99 | Gavage | D,G |
| PE 38:5 | 35.2 ± 1.84 | 33.3 ± 2.36 | 58.1 ± 3.78 | 59.6 ± 3.16 | 62.5 ± 3.89 | Diet | D |
| PE 38:4 | 52.8 ± 2.39 | 54.6 ± 2.36 | 64.9 ± 3.95 | 71.6 ± 4.07 | 43.8 ± 3.68 | Diet, Gavage | D |
| PE 38:3 | 8.04 ± 0.37 | 8.12 ± 0.58 | 9.45 ± 0.51 | 10.3 ± 0.60 | 6.89 ± 0.56 | Gavage | D |
| PE 38:2 | 0.76 ± 0.04 | 0.76 ± 0.06 | 1.01 ± 0.06 | 1.20 ± 0.08 | 1.16 ± 0.07 | Diet | D |
| PE 38:1 | 0.19 ± 0.01 | 0.22 ± 0.02 | 0.28 ± 0.01 | 0.37 ± 0.02 | 0.73 ± 0.04 | Diet, Gavage | D,G, DXG |
| PE 40:8 | 0.68 ± 0.03 | 0.88 ± 0.07 | 1.37 ± 0.10 | 1.73 ± 0.11 | 3.59 ± 0.22 | Diet, Gavage | D,G |
| PE 40:7 | 5.68 ± 0.29 | 7.81 ± 0.61 | 9.06 ± 0.66 | 13.02 ± 1.02 | 41.7 ± 2.67 | Gavage | D,G |
| PE 40:6 | 8.60 ± 0.45 | 10.33 ± 0.91 | 11.5 ± 0.83 | 14.9 ± 1.09 | 41.7 ± 2.67 | Gavage | D,G |
| PE 40:5 | 5.50 ± 0.29 | 5.25 ± 0.43 | 6.85 ± 0.51 | 7.06 ± 0.49 | 10.5 ± 0.66 | Gavage | D |
| PE 40:4 | 1.74 ± 0.09 | 1.77 ± 0.14 | 1.95 ± 0.09 | 2.07 ± 0.12 | 1.23 ± 0.07 | Gavage | D,G |
| PE 40:3 | 0.25 ± 0.01 | 0.26 ± 0.02 | 0.27 ± 0.01 | 0.30 ± 0.02 | 0.17 ± 0.01 | Gavage | ---- |
| PE O36:5 | 0.16 ± 0.01 | 0.18 ± 0.01 | 0.14 ± 0.01 | 0.16 ± 0.01 | 0.12 ± 0.01 | ----- | D,G |
| PE 36:0/O38:7 | 0.12 ± 0.01 | 0.11 ± 0.01 | 0.14 ± 0.01 | 0.16 ± 0.01 | 0.27 ± 0.02 | Gavage | D |
| PE 37:5/O38:5 | 0.23 ± 0.01 | 0.27 ± 0.02 | 0.35 ± 0.02 | 0.44 ± 0.02 | 0.34 ± 0.02 | Diet | D,G |
| PE 37:4/O38:4 | 0.39 ± 0.02 | 0.43 ± 0.04 | 0.45 ± 0.03 | 0.50 ± 0.03 | 0.31 ± 0.03 | Gavage | D |
| PE 39:4/O40:4 | 0.39 ± 0.02 | 0.48 ± 0.04 | 0.35 ± 0.02 | 0.40 ± 0.02 | 0.25 ± 0.01 | Gavage | D,G |

## Genetic model

**Total Ceramides**

**A** D: p≤.0001 G: p=.75 DxG: p=.76


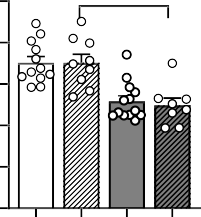
**10** ✱

**8**

**% Total Lipids**

**6**

**4**

**2**

**0**

**WT Fat-1 WT**

**Fat-1**

**LF LF HF HF**

# B


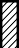

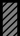

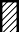

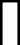

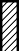

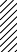

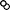

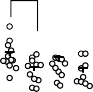

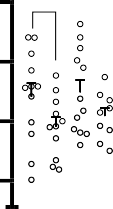

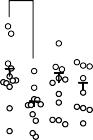

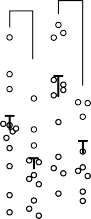

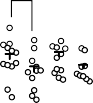

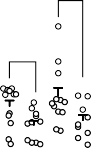

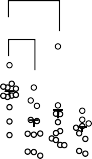

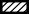

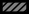


*p=.06*

Fat-1 LF WT HF Fat-1 HF

*p=.09*

*p=.08*

✱

✱

✱

✱✱

✱✱

✱

✱

**18000**

**13500**

**Liver cytokines**


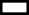
WT LF

**70000**


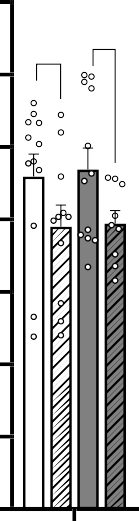


*p=.09*

*p=.08*

**60000**

**9000**

**4500**

**2500**

**pg/liver**

**2000**

**1500**

**1000**

**500**

**300**

**200**

**100**

**0**

**50000**

**40000**

**30000**

**20000**

**10000**

**0**

|  | **IL-1**β | **IL-2** | **IL-3** | **IL-6** | **IL-10** | **IFN-**γ | **RANTES** | **TNF-**α |
| --- | --- | --- | --- | --- | --- | --- | --- | --- |
| **Effect** | **G** | **G** | **G** | **G** | **G** | **D, G** | **G** | **G** |

**Supplemental Fig. S5**. **Impact of endogenous production of ω-3 PUFA on hepatic inflammation.** (**A**) Hepatic ceramides. (**B**) IL-1β, IL-2, IL-3, IL-6, IL-10, IFN-γ, RANTES and TNF-α hepatic concentrations. Values are means ± SEM of n=9-13 mice per group. Two- way ANOVA followed by Tukey *post hoc* test. If significant, main effects for diet (D) and gene (G) or diet x gene (DxG) interaction are indicated by the corresponding letter under the x axis for each inflammatory marker. *P<.05, **P<.01, ***P<.001.

**Dietary model**

# A

**Total Ceramides**

**10**


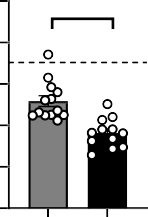


✱✱✱✱

**8**

**% Total Lipids**

**6**

**4**

**2**

**0**

**B 20000**


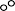

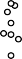

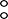

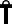

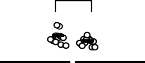

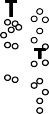

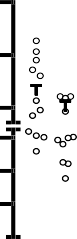

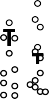

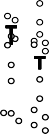

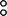

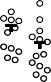

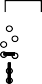

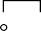


**15000**

**10000**

**8000**

**6000**

**4000**

**pg/liver**

**2000**

**1500**

**1000**

**500**

**HFω6 HFω3**

✱✱✱

✱

### Liver cytokines

HF ω6 HF ω3

✱

**70000**


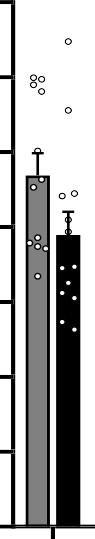
**60000**

**50000**

**40000**

**30000**

**20000**

**10000**

**0**

**IL-1**β **IL-2 IL-3 IL-6 IL-10 IFN-**γ **RANTES**

**0**

**TNF-**α

### Supplemental Fig. S6. Impact of ω-3 PUFA supplementation on hepatic inflammation.

(**A**). Hepatic ceramides. (**B**) IL-1β, IL-2, IL-3, IL-6, IL-10, IFN-γ,

RANTES and TNF-α hepatic concentrations. Values are means ± SEM of n=9-13 mice per group. One-way ANOVA followed by Dunnett *post hoc* test compared to HF-ω6 group. Dotted line represents LF-ω6 group as a reference. *P<.05, **P<.01, ***P<.001.

**Supplemental Table S4**. **Hepatic bile acid profile (pM/mg and %).** The two last columns show statistical differences referring to the dietary and the genetic models respectively. Dietary model: One-way ANOVA with Tukey *post hoc* test (*vs* HF ω6 group). Significant effects are notified by “diet” (LF WT ω6 *vs* HF ω6) or “gavage” (HF ω3 group *vs* HF ω6). Genetic model: 2-way ANOVA followed by a Tukey *post hoc* test. Significant main effects are notified by “D” (diet) or “G” (gene).

| **Hepatic Bile acids**  **(pM/mg)** | **LF WT ω6 LF Fat-1 ω6 HF WT ω6 HF Fat-1 ω6 HF WTω3** | | | | | **Dietary model Genetic model**  **One-way ANOVA listing 2-way ANOVA listing** | |
| --- | --- | --- | --- | --- | --- | --- | --- |
| **CA** | 0.99 ± 0.42 | 1.08 ± 0.42 | 0.15 ± 0.03 | 0.22 ± 0.06 | 0.52 ± 0.18 | diet, gavage | D |
| **TCA** | 75.14 ± 11.19 | 105.33 ± 16.51 | 103.44 ± 11.82 | 139.11 ± 26.01 | 129.97 ± 13.10 | - | D: p=.07 |
| **GCA** | 0.07 ± 0.01 | 0.11 ± 0.03 | 0.07 ± 0.02 | 0.10 ± 0.04 | 0.13 ± 0.02 | gavage | - |
| **CDCA** | 0.01 ± 0.00 | 0.01 ± 0.00 | 0.01 ± 0.00 | 0.00 ± 0.00 | 0.00 ± 0.00 | - | - |
| **TCDCA** | 6.07 ± 1.05 | 7.74 ± 1.25 | 8.81 ± 0.80 | 9.82 ± 1.19 | 8.43 ± 1.00 | diet | D |
| **αMCA** | 0.21 ± 0.07 | 0.36 ± 0.09 | 0.16 ± 0.04 | 0.13 ± 0.03 | 0.18 ± 0.04 | - | - |
| **TαMCA** | 27.88 ± 4.52 | 35.34 ± 4.17 | 32.43 ± 3.18 | 36.84 ± 6.77 | 36.51 ± 4.64 | - | - |
| **βMCA** | 1.00 ± 0.36 | 1.89 ± 0.53 | 1.00 ± 0.17 | 1.20 ± 0.39 | 1.42 ± 0.27 | - | - |
| **TβMCA** | 111.70 ± 35.7 | 156.44 ± 20.14 | 160.10 ± 16.98 | 201.02 ± 50.87 | 198.40 ± 24.96 | diet: p=.08 | D: p=.06, G: p=.07 |
| **UDCA** | 0.03 ± 0.01 | 0.03 ± 0.01 | 0.03 ± 0.01 | 0.01 ± 0.00 | 0.02 ± 0.01 | - | - |
| **TUDCA** | 8.75 ± 1.65 | 10.49 ± 1.28 | 11.71 ± 1.39 | 11.93 ± 1.92 | 10.42 ± 1.37 | - | - |
| **Total primary** | 231.86 ± 52.25 | 318.83 ± 39.53 | 317.92 ± 29.93 | 400.39 ± 80.70 | 386.00 ± 41.67 | - | D: p=.06, G: p=.07 |
| **DCA** | 0.00 ± 0.00 | 0.00 ± 0.00 | 0.07 ± 0.05 | 0.01 ± 0.00 | 0.01 ± 0.01 | diet | - |
| **TDCA** | 2.09 ± 0.67 | 4.16 ± 1.32 | 4.02 ± 1.13 | 4.45 ± 1.92 | 2.21 ± 0.36 | - | - |
| **TLCA** | 0.03 ± 0.01 | 0.05 ± 0.01 | 0.04 ± 0.01 | 0.03 ± 0.01 | 0.03 ± 0.00 | - | - |
| **ωMCA** | 0.44 ± 0.07 | 1.06 ± 0.29 | 0.32 ± 0.09 | 0.27 ± 0.06 | 0.56 ± 0.19 | - | D |
| **TωMCA** | 70.03 ± 7.04 | 96.19 ± 18.23 | 47.17 ± 10.43 | 56.01 ± 6.70 | 70.73 ± 10.52 | - | D |
| **Total secondary** | 72.60 ± 7.39 | 101.48 ± 18.64 | 51.63 ± 10.5 | 60.77 ± 8.35 | 73.54 ± 10.51 | - | D |
| **Total BA** | 304.46 ± 54.12 | 420.31 ± 49.59 | 369.55 ± 36.73 | 461.16 ± 81.54 | 459.53 ± 44.13 | - | G: p=.07 |
| **Primary / secondary** | 3.33 ± 0.69 | 4.57 ± 1.04 | 10.06 ± 2.43 | 7.57 ± 2.13 | 6.60 ± 1.35 | diet | D |
| **Total conjugated** | 301.77 ± 53.56 | 415.86 ± 48.89 | 367.80 ± 36.44 | 459.32 ± 81.21 | 456.81 ± 43.97 | - | G: p=.07 |
| **Total unconjugated** | 2.69 ± 0.85 | 4.44 ± 1.09 | 1.75 ± 0.34 | 1.84 ± 0.51 | 2.72 ± 0.58 | Gavage: p=.09 | - |
| **Conjugated / unconjugated** | 171.87 ± 26.42 | 220.62 ± 82.83 | 248.10 ± 26.16 | 351.28 ± 61.69 | 216.64 ± 32.36 | - | D |
| **Hepatic Bile acids (% of BA pool)** | **LF WT ω6 LF Fat-1 ω6 HF WT ω6 HF Fat-1 ω6 HF WTω3** | | | | | **Dietary model Genetic model One-way ANOVA listing Two-way ANOVA listing** | |
| **CA** | 0.29 ± 0.09 | 0.24 ± 0.1 | 0.04 ± 0.01 | 0.04 ± 0.01 | 0.11 ± 0.03 | Diet, gavage | D |
| **TCA** | 25.43 ± 1.30 | 24.27 ± 1.96 | 28.1 ± 1.86 | 29.74 ± 2.01 | 28.37 ± 1.27 | - | D |
| **GCA** | 0.02 ± 0.00 | 0.02 ± 0.00 | 0.02 ± 0.00 | 0.02 ± 0.01 | 0.03 ± 0.00 | - | - |
| **CDCA** | 0.00 ± 0.00 | 0.00 ± 0.00 | 0.00 ± 0.00 | 0.00 ± 0.00 | 0.00 ± 0.00 | - | - |
| **TCDCA** | 2.04 ± 0.10 | 1.88 ± 0.19 | 2.51 ± 0.22 | 2.39 ± 0.24 | 1.88 ± 0.13 | Gavage: p=.05 | D |
| **αMCA** | 0.07 ± 0.02 | 0.08 ± 0.02 | 0.04 ± 0.01 | 0.03 ± 0.00 | 0.04 ± 0.01 | - | D |
| **TαMCA** | 9.39 ± 0.37 | 8.56 ± 0.50 | 8.83 ± 0.34 | 8.21 ± 0.54 | 7.75 ± 0.39 | - | - |
| **βMCA** | 0.28 ± 0.07 | 0.41 ± 0.08 | 0.26 ± 0.03 | 0.23 ± 0.06 | 0.31 ± 0.04 | - | - |
| **TβMCA** | 31.76 ± 3.35 | 38.45 ± 3.82 | 43.37 ± 2.91 | 40.76 ± 3.43 | 41.94 ± 2.44 | diet | D |
| **UDCA** | 0.01 ± 0.00 | 0.01 ± 0.00 | 0.01 ± 0.00 | 0.00 ± 0.00 | 0.01 ± 0.00 | - | - |
| **TUDCA** | 2.93 ± 0.27 | 2.62 ± 0.25 | 3.25 ± 0.33 | 2.69 ± 0.18 | 2.21 ± 0.15 | gavage | - |
| **Total primary** | 72.22 ± 3.18 | 76.54 ± 3.54 | 86.44 ± 2.06 | 84.13 ± 2.23 | 82.66 ± 2.20 | diet | D |
| **DCA** | 0.00 ± 0.00 | 0.00 ± 0.00 | 0.01 ± 0.01 | 0.00 ± 0.00 | 0.01 ± 0.01 | diet | - |
| **TDCA** | 0.86 ± 0.26 | 0.96 ± 0.28 | 1.12 ± 0.32 | 0.94 ± 0.37 | 0.54 ± 0.09 | - | - |
| **TLCA** | 0.01 ± 0.00 | 0.01 ± 0.00 | 0.01 ± 0.00 | 0.01 ± 0.00 | 0.01 ± 0.00 | - | - |
| **ωMCA** | 0.16 ± 0.02 | 0.24 ± 0.06 | 0.08 ± 0.02 | 0.07 ± 0.01 | 0.22 ± 0.14 | diet | D |
| **TωMCA** | 26.75 ± 3.03 | 22.24 ± 3.6 | 12.33 ± 2.04 | 14.86 ± 2.15 | 16.56 ± 2.08 | diet | D |
| **Total secondary** | 27.78 ± 3.18 | 23.46 ± 3.54 | 13.56 ± 2.06 | 15.87 ± 2.23 | 17.34 ± 2.20 | diet | D |
| **Total conjugated** | 99.19 ± 0.17 | 99.02 ± 0.21 | 99.54 ± 0.05 | 99.62 ± 0.07 | 99.29 ± 0.20 | - | D |
| **Total unconjugated** | 0.81 ± 0.17 | 0.98 ± 0.21 | 0.46 ± 0.05 | 0.38 ± 0.07 | 0.71 ± 0.20 | - | D |

**Genetic model**

**D**

**E**

**A Palmitic acid B**

**C16:0**

**2-PG C**

**PEA**

**Oleic acid C18:1n9**

**2-OG**

1. **OEA**

**D: p=.01 G: p=.07** DxG: p=.60

D: p=.95 **G: p=.01** DxG: p=.26

**D: p<.001 G: p=.01 DxG: p=.005 D: p=.002 G: p=.03** DxG: p=.60

D: p=.15 **G: p=.002 DxG: p=.04**

**D: p<.001 G: p=.02** DxG: p=.89


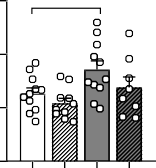
**3000** ✱ **3**


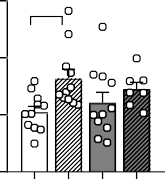


✱✱

**2000 2**

**µM**

**pmol/mg liver**

**0.25**

**0.20**

**pmol/mg liver**

**0.15**

✱✱✱

✱✱✱

✱✱

**4500 30**


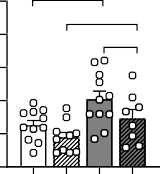


*p=.07*

*p=.06*


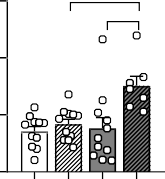


✱

✱✱

**3600**

**pmol/mg liver**

**20**

**2700**

**µM**

**0.3** ✱✱

**0.2**


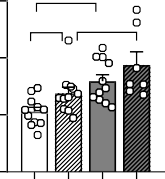


*p=.06* ✱

**pmol/mg liver**

**1000 1**

**0.10**

**0.05**

**1800**

**10**

**900**

**0.1**

**0**

**WT Fat-1 WT Fat-1**

**0**

**WT Fat-1 WT Fat-1**

**0.00**


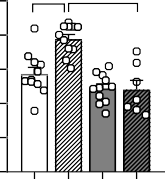
**WT Fat-1 WT Fat-1**

**0**

**WT Fat-1 WT Fat-1**

**0**

**WT Fat-1 WT Fat-1**

**0.0**

**WT Fat-1 WT Fat-1**

**LF LF**

**HF HF**

**LF LF**

**HF HF**

**LF LF**

**HF HF**

**LF LF**

**HF HF**

**LF LF**

**HF HF**

**LF LF**

**HF HF**

## Dietary model

1. **Palmitic acid H**

**C16:0**

**2-PG I**

**PEA**

**Oleic acid C18:1n9**

**2-OG**

**M OEA**

**3**

**K**

**L**

**3000** ✱✱


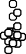


**2000 2**

**µM**

**pmol/mg liver**

**1000 1**

**0.20**

**0.15**

**pmol/mg liver**

**0.10**

**0.05**

**4000**

**3000**

**2000**

**µM**

**1000**

✱✱✱ **25**

**20**


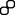

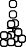


**pmol/mg liver**

**15**

**10**

**5**

**0.25**

**0.20**

**pmol/mg liver**

**0.15**

**0.10**

**0.05**

**0**

**HF HF**

**0**

**HF HF**

**0.00**

**HF HF**

**0**

**HF HF**

**0**

**HF HF**

**0.00**

**HF HF**

ω**6** ω**3**

ω**6** ω**3**

ω**6** ω**3**

ω**6** ω**3**

ω**6** ω**3**

ω**6** ω**3**

## Genetic model Dietary model

**N *Cnr1***

D: p=.49 G: p=.72 **DxG: p=.05**

**Normalized Expression (Log2)**


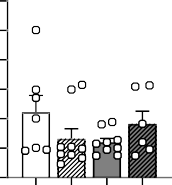
**3.0**

**2.5**

**2.0**

**1.5**

**1.0**

**0.5**

**0.0**

**O**

**2.0**

**Normalized Expression (Log2)**

**1.5**

**1.0**

**0.5**

**0.0**

***Cnr1***

**WT Fat-1 WT Fat-1 HF HF**

**LF LF**

**HF HF**

ω**6** ω**3**

**Supplementary Fig S7. Impact of endogenous production and supplementation of ω-3 PUFA on hepatic endocannabinoids.** Panels (**A-F, N**) refer to the genetic model and (**G-M, O**) to the dietary model. (**A, G**) Palmitic acid and its (**B, H**) 2- monoacylglycerol and (**C, I**) N-acylethanolamine forms. (**D, K**) Oleic acid and its (**E, L**) 2-monoacylglycerol and (**F, M**) N- acylethanolamine forms. (**N-O**) Relative expression of *Cnr1* coding for the cannabinoid receptor type-1 (CB_1_) in liver in (**N**) the genetic model and (**O**) the dietary model. Values are means ± SEM of n=9-13 mice per group. For the genetic model: two-way ANOVA followed by Tukey post hoc test. P-values of main effects for diet (D) and gene (G) or diet x gene (DxG) interaction are recorded under the title of each graph. For the dietary model: one-way ANOVA followed by Dunnett post hoc test compared to HF-ω6 group. Dotted line represents LF-ω6 group as a reference. *P<.05,

**P<.01, ***P<.001.

## Genetic model

**2-EPG**

**A**

**B**

**D: p=.07 G: p<.001 DxG: p=.05**

**EPEA**

**D: p=.09 G: p<.001 DxG: p=.05**

**2-DPG**

D: p=.65 **G: p<001 DxG: p=.004**

**C**

**2-DHG**

**D: p=.07 G: p<.001** DxG: p=.11

**D**

**E**

**DHEA**

D: p=.47 **G: p=.002** DxG: p=.90

**0.12**

**0.09**

**pmol/mg gastroc**

**0.06**

**0.03**

**0.00**

**WT Fat-1 WT Fat-1**

**0.0012**

**0.0009**


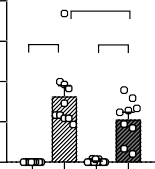


✱

✱✱✱

✱✱✱

**pmol/mg gastroc**

**0.0006**

**0.0003**

**0.0000**

**WT Fat-1 WT Fat-1**

**0.6**

**0.5**


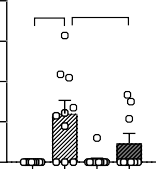


✱✱✱ ✱

**pmol/mg gastroc**

**0.4**

**0.3**

**0.2**

**0.1**

**0.0**

*p=.09*

**WT Fat-1 WT Fat-1**


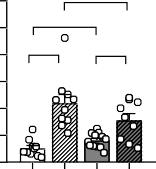


✱

✱✱✱

✱

**2.0**

**1.5**

**pmol/mg gastroc**

**1.0**

**0.5**

**0.0**

**WT Fat-1 WT Fat-1**

**0.015**

**0.010**


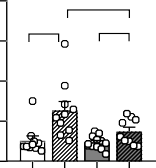


✱

✱✱✱

✱

**pmol/mg gastroc**

**0.005**

**0.000**

✱ ✱

**WT Fat-1 WT Fat-1**

**LF LF HF HF**

**2-LG**

**LF LF HF HF**

**LEA**

**LF LF HF HF**

**2-AG**

**LF LF HF HF**

**AEA**

**LF LF HF HF**

**F** D: p=.44 **G: p=.03 DxG: p=.05**

**G** D: p=.32 G: p=.16 DxG: p=.66

**H** D: p=.52 **G: p=.002 DxG: p=.01 I**

D: p=.16 G: p=.25 DxG: p=.34

**15**

✱

✱✱

**pmol/mg gastroc**

**10**

**5**

**0**

**WT Fat-1 WT Fat-1**

**0.020**

**0.015**

**pmol/mg gastroc**

**0.010**

**0.005**

**0.000**

**1.5** ✱

**1.0**

**pmol/mg gastroc**

**0.5**

**0.0**

**0.006**

**0.004**

✱✱✱

**pmol/mg gastroc**

**0.002**

**0.000**

**WT Fat-1 WT Fat-1**

**LF LF HF HF**

**WT Fat-1 WT Fat-1**

**WT Fat-1 WT Fat-1**

**LF LF HF HF**

**2-PG**

**LF LF HF HF**

**LF LF HF HF**

**J** D: p=.18 G: p=.28 **DxG: p=.07**

**PEA**

D: p=.65 G: p=.84 DxG: p=.87

**K**

**2-OG**

D: p=.42 **G: p=.03 DxG: p=.08**

✱✱

*p=.09* ✱✱✱

**L**

**OEA**

**D: p<.001** G: p=.16 DxG: p=.30

**6** ✱ ✱

**pmol/mg gastroc**

**4**

**0.20 6**

**0.15**

**pmol/mg gastroc**

**pmol/mg gastroc**

**4**

*p=.06*

**M 0.3** ✱✱

**0.2**

**pmol/mg gastroc**

**2**

**0**

**WT Fat-1 WT Fat-1**

**0.10**

**0.05**

**0.00**

**2**

**0**

**WT Fat-1 WT Fat-1**

**0.1**

**0.0**

**WT Fat-1 WT Fat-1**

**LF LF HF HF**

**WT Fat-1 WT Fat-1**

**LF LF HF HF**

**LF LF HF HF**

**LF LF HF HF**

**N 0.8**

**0.6**

**pmol/mg gastroc**

**0.4**

**0.2**

**2-EPG**

**O 0.003**

✱✱✱

**pmol/mg gastroc**

**0.002**

**0.001**

**EPEA**

✱✱✱

## Dietary model

**2-DPG**

**P**

**Q**

✱✱

**1.5**

**pmol/mg gastroc**

**1.0**

**0.5**

**2-DHG**

✱✱✱

**40**

**30**

**pmol/mg gastroc**

**20**

**10**

**R 0.015**

**0.010**

**pmol/mg gastroc**

**0.005**

**DHEA**

✱✱✱

**0.0**

**HF HF**

ω**6** ω**3**

**S T**

**2-LG**

**0.000**

**HF HF**

ω**6** ω**3**

**LEA**

**0.0**

**HF HF**

ω**6** ω**3**

**U**

**2-AG**

**0**

**HF HF**

ω**6** ω**3**

**V**

**AEA**

✱✱✱

**0.000**

**HF HF**

ω**6** ω**3**

**15**

✱✱

**pmol/mg gastroc**

**10**

**5**

**0**

**HF HF**

**0.008**

**0.006**

**pmol/mg gastroc**

**0.004**

**0.002**

**0.000**

✱✱✱

**HF HF**

**0.6**

**0.4**

**pmol/mg gastroc**

**0.2**

**0.0**

✱✱✱

**HF HF**

**0.004**

**0.003**

**pmol/mg gastroc**

**0.002**

**0.001**

**0.000**

**HF HF**

ω**6** ω**3**

ω**6** ω**3**

ω**6** ω**3**

ω**6** ω**3**

**W 2-PG**

**6** *p=.07*

**pmol/mg gastroc**

**4**

**2**

**0**

**HF HF**

**X**

**0.20**

**0.15**

**pmol/mg gastroc**

**0.10**

**0.05**

**0.00**

**PEA**

**HF HF**

**Y 2-OG**

**6**

**pmol/mg gastroc**

**4**

**2**

1.6327

**0**

**HF HF**

**Z**

**0.10**

**0.08**

**pmol/mg gastroc**

**0.06**

**0.04**

**0.02**

**0.00**

**OEA**

**HF HF**

ω**6** ω**3**

ω**6** ω**3**

ω**6** ω**3**

ω**6** ω**3**

**Supplementary Fig. S8. Impact of endogenous production and supplementation of ω-3 PUFA on endocannabinoid profile in the gastrocnemius muscle.** Panels (**A-M**) refer to the genetic model and (**N-Z**) to the dietary model. (**A, N**) 2-MAG and (**B, O**) NAE forms of EPA. (**C, P**) 2-MAG form of n-3 DPA. (**D, Q**) 2-MAG and (**E, R**) NAE forms of DHA. (**F, S**) 2-MAG and (**G, T**) NAE forms of LA. (**H, U**) 2-MAG and (**I, V**) NAE forms of AA. (**J, W**) 2-MAG and (**K, X**) NAE forms of palmitic acid. (**L, Y**) 2-MAG and (**M, Z**) NAE forms of oleic acid. Values are means ± SEM of n=9-13 mice per group. For the genetic model: two- way ANOVA followed by Tukey post hoc test. P-values of main effects for diet (D) and gene (G) or diet x gene (DxG) interaction are recorded under the title of each graph. For the dietary model: one-way ANOVA followed by Dunnett post hoc test compared to HF-ω6 group. Dotted line represents LF-ω6 group as a reference. *P<.05, **P<.01, ***P<.001.

### Supplementary Table S5. Quantitative variables were grouped into 11 factors for multiple facator analysis.

**Group Definitions**

**Genetic** Wildtype(WT) **Diet** low fat diet (LF)

Fat1 genotype(F1) High fat diet (HF)

HF diet + omega3 supplementtion (HF-w3)

| **Factors** |  | | | | | | |
| --- | --- | --- | --- | --- | --- | --- | --- |
| **Obesity** | Body weight gain (BWG) | **Endocanom** | liver EPA2G(μM)(LIV_EPA2G) | **Microbiota** | s_Other | **Gluco_Home** | Fastin blood glucose T0 (FG_GTT) |
|  | Food Intake (FI) |  | liver t DHA2G(μM)(LIV_DHA2G) |  | g Bifidobacterium_s |  | Fastin blood insulin T0 (FI_GTT) |
|  | Energy expenditure(EE) |  | liver DPA2G(μM)(LIV_DPA2G) |  | g Adlercreutzia_s |  | Area under the curve for glucose during ITT (AUC_ITT) |
|  | DEE |  | liver EPEA(μM)(LIV_EPEA) |  | g Barnesiella_s |  | Area under the curve for glucose during GTT (AUC_GTT) |
|  | Visceral adipose weight(Vat_W) |  | liver DHEA(μM)(LIV_DHEA) |  | g Parabacteroides_s |  | Area under the curve glucose stimulated insulin GTT (AUC_GSIS) |
|  | Plasma leptin concentration (Leptin) |  | muscle EPEA(μM)(MUS_EPEA) |  | f Rikenellaceae_g_s |  | HOMA-IR |
|  |  |  | muscle c DHEA(μM)(MUS_DHEA) |  | f S247_g_s |  |  |
| **NAFLD** | plasma ALT (P_ALT)  plasma AST (P_AST) |  | muscle EPA2G(μM)(MUS_EPA2G)  muscle DHA2G(μM)(MUS_DHA2G) |  | s schaedleri  g Lactococcus_s | **Intestinal**  **Metabolism** | caecal acetic acid (μg/g)(Acetic_acid) |
|  | liver weight (LIV_W) liver TG (LIV_TG)  Total liver fatty acid (μM) (LIV_FA)  SCD1 index (SCD1) SCD16 index (SCD16) |  | muscle DPA2G(μM)(MUS_ DPA2G) liver AEA(μM)(LIV_AEA)  liver 2AG(μM)(LIV_2AG) liver 2LG(μM)(LIV_2LG)  liver LEA(μM)(LIV_LEA) |  | g Streptococcus_s g Turicibacter_s   - Clostridiales_f_g_s - Clostridiales_f_g_s_2   f Christensenellaceae_g_s |  | caecal Propionic acid (μg/g)(Propionic_acid) caecal butyric acid (μg/g)(Butyric_acid) caecal valeric acid (μg/g)(vlaeric_acid)  caecal isobutyric acid (μg/g)(isobutyric_acid)  caecal isovaleric acid (μg/g)(isovaleric_acid) |
|  | SCD18 index (SCD18) |  | muscle AEA(μM)(MUS_AEA) |  | f Clostridiaceae_g_s |  | caecum weight (g) (caecum) |
|  | Liver w6:w3 ratio (LIV_W6W3) Liver w3 (μM) (LIV_W3)  Liver w6 (μM) (LIV_W6) |  | muscle 2AG(μM)(MUS_2AG)  muscler 2LG(μM)(MUS_2LG) muscler LEA(μM)(MUS_LEA) |  | g Clostridium_s g Anaerofustis_s  f Lachnospiraceae_g_s |  | feces LCN2 (ng/g)(FLCN2) feces LPS (μg/g)(Feces_LPS)  feces flagellin (ng/mg)(Feces_Flagellin) |
|  |  |  | liver 2PG(μM)(LIV_2PG) |  | f Lachnospiraceae_g_s_2 |  | liver CA (pM/mg)(CA) |
| **FA_PUFA** | liver EPA(μM)(LIV_EPA) |  | liver PEA(μM)(LIV_PEA) |  | g Anaerostipes_s |  | liver TCA (pM/mg)(TCA) |
|  | liver DHA(μM)(LIV_DHA) |  | liver OEA(μM)(LIV_OEA) |  | g Blautia_s |  | liver GCA (pM/mg)(GCA) |
|  | liver DPA(μM)(LIV_DPA) |  | liver 2OG(μM)(LIV_2OG) |  | g Coprococcus_s |  | liver CDCA (pM/mg)(CDCA) |
|  | Liver AA(μM) (LIV_AA) |  | muscle PEA(μM)(MUS_PEA) |  | g Dorea_s |  | liver TCDCA (pM/mg)(TCDCA) |
|  | Liver LA(μM) (LIV_LA) |  | muscle 2PG(μM)(MUS_2PG) |  | s gnavus |  | liver aMCA (pM/mg)(aMCA) |
|  |  |  | muscler 2OG(μM)(MUS_2OG) |  | f Ruminococcaceae_g_s |  | liver TaMCA (pM/mg)(TaMCA) |
| **FA_SAT** | liver capric acid (μM)(Caprique) |  | muscler OEA(μM)(MUS_OEA) |  | f Ruminococcaceae_g_s_2 |  | liver bMCA (pM/mg)(bMCA) |
|  | liver myristic acid (μM)(Myristique) |  |  |  | g Oscillibacter_s |  | liver TbMCA (pM/mg)(TbMCA) |
|  | liver palmitic acid (μM)(Palmitique) | **Plasma** | Plasma TG (P_TG) |  | g Oscillospira_s |  | liver UDCA (pM/mg)(UDCA) |
|  | liver stearic acid (μM)(Stearique) |  | Plasma Nonesterfied fatty acids(P_NEFA) |  | g Ruminococcus_s |  | liver TUDCA (pM/mg)(TUDCA) |
|  | liver arachidonic acid (μM)(Arachidique) |  | Plasma cholesterol (P_Chol) |  | f [Mogibacteriaceae]_g_s |  | liver total primary bile acids (pM/mg)(Totprim) |
|  | liver Behenic acid (μM)(Behebique) |  |  |  | f Erysipelotrichaceae_g_s |  | liver DCA (pM/mg)(DCA) |
|  | liver lignoceric acid (μM)(Lignocerique) | **Inflammation** | liver LTB4(pg/mg)(LIV_LTB4) |  | g Allobaculum_s |  | liver TDCA (pM/mg)(TDCA) |
|  | liver myristoleic acid (μM)(myristoleic) |  | liver 17S (pg/mg)(LIV_17S) |  | g Coprobacillus_s |  | liver TLCA (pM/mg)(TLCA) |
|  | liver pamitoleic acid (μM)(palmitoleic) |  | liver 18RS (pg/mg)(LIV_18RS) |  | s C21_c20 |  | liver wMCA (pM/mg)(wMCA) |
|  | liver vaccenic acid (μM)(vaccenic) |  | muscle LTB4(pg/mg)(musc_LTB4) |  | g Proteus_s |  | liver TwCA (pM/mg)(TwCA) |
|  |  |  | muscle17S (pg/mg)(mus_17S) |  | g Anaeroplasma_s |  | liver total secondary bile acids (pM/mg)(Totsec) |
| **FA_Unsat** | liver oleic acid (μM)(oleic)  liver petroselinic acid (μM)(petroselinic) |  | muscle 18RS (pg/mg)(mus_18RS)  liver IL-1β(pg/liver)(IL-1β) |  | s muciniphila |  | liver total conjugated bile acids (pM/mg)(TotCon)  liver total primary bile acids (pM/mg)(TotUcon) |
|  | liver n9 eicosenoic acid (μM)(eicosenoic_9) |  | liver IL2(pg/liver)(IL2) |  |  |  |  |
|  | liver n12 eicosenoic acid (μM)(eicosenoic_12) |  | liver IL3(pg/liver)(IL3) |  |  |  |  |
|  | liver n15 eicosenoic acid (μM)(eicosenoic_15) |  | liver IL6(pg/liver)(IL6) |  |  |  |  |
|  | liver erucic acid (μM)(erucic)  liver nervonic acid (μM)(nervonic) |  | liver IL10 (pg/liver)(IL10)  liver IFNγ (pg/liver)(IIFNγ) |  |  |  |  |
|  | liver palmitelaiditic acid (μM)(Palmitelaiditic)  liver Transvaccenicc acid (μM)(Transvaccenic) |  | liver RANTES (pg/liver)(RANTES)  liver TNFα (pg/liver)(TNFα) |  |  |  |  |
|  | liver elaiditic acid (μM)(elaiditic)  liver γlinolenic acid (μM)(linolenic) |  |  |  |  |  |  |
|  | liver eicosadienoicacid (μM)(eicosadienoic)  liver DHGLinolenic acid (μM)(DHGLinolenic) |  |  |  |  |  |  |
|  | liver docosadienoic acid (μM)(docosadienoic) |  |  |  |  |  |  |
|  | liver adrenic acid (μM)(adrenic) |  |  |  |  |  |  |
|  | liver n3-eicosatrienoic acid (μM)(eicosatrienoic3) |  |  |  |  |  |  |
|  | liver n9-eicosatrienoic acid (μM)(eicosatrienoic9) |  |  |  |  |  |  |

### Supplementary Table S6 Primers used for real-time qPCR.
